# Psychosocial factors associated with pain in spinal cord injury: a systematic review and meta-analysis

**DOI:** 10.1016/j.eclinm.2026.103976

**Published:** 2026-05-18

**Authors:** Negin Hesam-Shariati, Yann Quidé, Tong En Lim, Michael A. Wewege, Harrison J. Hansford, Thiago Folly de Campos, Yan Li, Lara Alexander, Toby Newton-John, James H. McAuley, Zina Trost, Sylvia M. Gustin

**Affiliations:** a NeuroRecovery Research Hub, School of Psychology, University of New South Wales, Sydney, NSW, Australia; b Centre for Pain IMPACT, Neuroscience Research Australia, Sydney, NSW, Australia; c School of Health Sciences, Faculty of Medicine and Health, University of New South Wales, Sydney, NSW, Australia; d School of Nursing, The Hong Kong Polytechnic University, Hung Hom, Hong Kong; e Graduate School of Health, University of Technology Sydney, Sydney, NSW, Australia; f Department of Psychological and Brain Sciences, Texas A&M University, College Station, TX, USA

**Keywords:** Spinal cord injury, Pain, Psychosocial, Meta-analysis

## Abstract

**Background:**

Spinal cord injury (SCI) affects over 20 million people worldwide, and pain is the most prevalent secondary condition with substantial psychosocial consequences. However, evidence on these consequences remains fragmented, limiting theoretical and practical integration. The aim of this study was to synthesise evidence on associations between pain intensity and psychosocial outcomes in adults with chronic SCI.

**Methods:**

CINAHL, Embase, PubMed, and Web of Science were searched from inception to 8 March 2026. Peer-reviewed studies were included if they reported associations between pain intensity and psychosocial factors in adults with chronic SCI (≥1 year post-injury). Screening and data extraction were conducted in duplicate in accordance with systematic review and meta-analysis guidelines. Random-effects models were used for all meta-analyses. Correlations were synthesised using Fisher's *z* transformation, and meta-regressions examined potential moderators. The review protocol was registered in PROSPERO (CRD42021276170).

**Findings:**

78 studies (n = 19,785 participants) were included. Most were cross-sectional, with sample sizes ranging from nine to nearly 5000 participants. Study quality was predominantly moderate, with seven high-quality and six low-quality reports. Moderate positive correlations were found between pain and depression (*r* 0.34, 95% CI 0.30 to 0.38), anxiety (0.34, 95% CI 0.28 to 0.40), catastrophising (0.37, 95% CI 0.29 to 0.44), anger (0.34, 95% CI 0.17 to 0.49), and fatigue (0.43, 95% CI 0.31 to 0.53). Moderate negative correlations were observed with psychological health (−0.30, 95% CI −0.36 to −0.24), self-efficacy (−0.29, 95% CI −0.38 to −0.20), acceptance (−0.27, 95% CI −0.38 to −0.16), resilience (−0.27, 95% CI −0.29 to −0.24), social functioning (−0.28, 95% CI −0.34 to −0.22), and quality of life/life satisfaction (−0.29, 95% CI −0.36 to −0.22). Meta-regression analyses showed limited and inconsistent moderation, with most tested moderators not significantly influencing associations. Sensitivity analyses supported the robustness of findings.

**Interpretation:**

This review provides a comprehensive synthesis of psychosocial correlates of SCI-related pain. Despite limitations related to cross-sectional designs and heterogeneity in measures, findings highlight the importance of integrating psychosocial processes into SCI pain management and support the development of more tailored interventions.

**Funding:**

Craig H. 10.13039/100005191Neilsen Foundation, Rebecca L. Cooper Medical Research Foundation, and 10.13039/501100009287NSW Health.

**Research in context**

**Evidence before this study**

We searched CINAHL, Embase, PubMed, and Web of Science from database inception to 24 January 2025, with an updated search conducted on 8 March 2026. Search terms included combinations of spinal cord injury, pain, and psychosocial factors. Eligible studies reported associations between pain intensity and at least one psychosocial outcome in people with spinal cord injury (SCI). Existing studies show that pain intensity is associated with several psychosocial factors in SCI, particularly depression, anxiety, and catastrophising. However, previous reviews have been limited in scope, typically focusing on a small number of constructs, lacking quantitative synthesis across domains, and not examining potential moderators. Most available evidence is derived from cross-sectional studies of variable methodological quality, with inconsistent reporting of injury and pain characteristics. No previous meta-analysis has systematically and comprehensively synthesised associations between pain intensity and a broad range of psychosocial factors in chronic SCI.

**Added value of this study**

This systematic review and meta-analysis synthesised 78 studies (n = 19,785 participants), providing the most comprehensive quantitative synthesis to date of associations between pain intensity and psychosocial factors in chronic SCI. Inclusion of additional data obtained directly from study authors enabled broader coverage than analyses restricted to reported correlations alone. By integrating evidence across multiple domains, including mental health, adaptive psychological factors, cognitive and emotional processes, social factors, fatigue and sleep, and quality of life, we identified consistent, moderate associations between pain and several key psychosocial constructs. We also examined the influence of demographic and injury-related characteristics, identifying sex, age, time since injury, and tetraplegia, as moderators of several associations, although their effects were limited and inconsistent.

**Implications of all the available evidence**

Combined with existing evidence, these findings support a (bio)psychosocial model of pain in SCI, highlighting the importance of psychological and social processes alongside neurobiological mechanisms. These findings highlight modifiable targets such as depression, catastrophising, and self-efficacy that may inform psychologically informed interventions and multidisciplinary rehabilitation approaches. Future research should prioritise longitudinal and interventional studies to clarify causal pathways and evaluate targeted interventions. Complementary evidence syntheses focusing on temporal dynamics and treatment effects may further advance understanding of mechanisms and optimise care for individuals with SCI.

## Introduction

Spinal cord injury (SCI) affects more than 20 million individuals worldwide, with prevalence increasing by more than 80% between 1990 and 2019.^1^^,^^2^ Pain is among the most common and disabling secondary health conditions following SCI,^3^ affecting approximately 50%–80% of individuals^4^^,^^5^ and contributing substantially to long-term disability and reduced quality of life.^3^ Although both pharmacological and non-pharmacological options are available, SCI-related pain often remains resistant to treatment,^6^ with benefits typically partial, temporary, or limited by adverse effects, cost, or restricted access.^7^, ^8^, ^9^ These limitations highlight the need to better understand factors associated with persistent pain beyond biomedical mechanisms alone.

In addition to neurological injury processes, psychosocial factors, such as psychological distress, coping responses, and social functioning, may influence pain intensity and persistence in individuals with SCI.^10^^,^^11^ Previous research suggests that these factors can shape pain experience independent of physiological mechanisms and may interact with individual- and injury-related characteristics, including age, sex, time since injury, and level or completeness of injury, either directly or through their interaction with other psychosocial comorbidities.^12^, ^13^, ^14^, ^15^ However, findings across studies are fragmented, and the relative importance of specific psychosocial factors remains unclear, limiting their integration into clinical assessment and intervention planning.

A comprehensive synthesis of psychosocial correlates of pain in adults with SCI is needed to clarify which factors are most consistently associated with pain intensity and to identify potential moderators. Accordingly, this systematic review synthesises evidence on psychosocial factors associated with pain intensity in adults with chronic SCI and, where data permit, examines whether individual- or injury-related characteristics modify these associations. This review aims to inform future research and multidisciplinary approaches to pain management in SCI.

## Methods

This study is a systematic review and meta-analysis of previously published studies and did not involve the collection of new data from human participants; therefore, ethics approval was not required. The review was reported in accordance with the Preferred Reporting Items for Systematic Reviews and Meta-Analyses (PRISMA) 2020 statement,^16^ and the review protocol was registered in PROSPERO (CRD42021276170).

### Search strategy and selection criteria

Four electronic databases (CINAHL, Embase, PubMed, and Web of Science) were systematically searched on 24 January 2025, and updated on 8 March 2026, to identify relevant peer-reviewed studies. Inclusion criteria were limited to studies conducted on humans and published in English, with no restrictions on the publication year. Search strategies combined keywords and MeSH terms related to spinal cord injury, pain, and psychosocial factors and were developed for each database (full strategies in Appendix 1).

Peer-reviewed original quantitative research studies were included, whereas reviews, dissertations, conference abstracts, and editorials were excluded. Eligible studies examined the association between pain intensity and psychosocial variables in individuals with chronic SCI. Observational (e.g., cross-sectional, longitudinal) and experimental studies (e.g., randomised or non-randomised trials) were included. If studies assessed pain intensity and psychosocial outcomes but did not report the correlations, authors were contacted up to three times to request raw data, if the data were unavailable, the study was excluded. Studies using binary (0/1) pain measures did not consistently group participants into with and without pain categories and lacked sufficient detail for cross-study comparability. We therefore excluded such studies and restricted analyses to those reporting pain intensity on continuous or ordinal scales.

Eligible studies included individuals aged 18 years or older with SCI of at least one year in duration. The original PROSPERO protocol required participants to have chronic pain, defined as persistent or recurrent pain lasting for at least three months.^17^ However, piloting this criterion revealed that many studies did not report pain duration. Authors were contacted to clarify the pain chronicity, but this information was often unavailable. Therefore, studies with ambiguous pain duration were included if participants had an SCI duration of at least one year, acknowledging the high prevalence of chronic pain in this population.

Eligible studies assessed pain intensity and at least one psychosocial outcome (e.g., depression, resilience, social functioning, or quality of life). Pain intensity was assessed using validated self-report instruments, including but not limited to the numerical rating scale (NRS), the visual analogue scale (VAS), the Brief Pain Inventory (BPI). Studies were eligible if pain intensity was defined as average pain over a specified recall period (e.g., past 24 h or past week), current pain, or global pain intensity. Psychosocial outcomes were included if they represented conceptually comparable constructs that could be synthesised within each meta-analysis. Variability in rating scales and recall periods for these outcomes did not constitute an exclusion criterion.

### Screening

All records from the searches were imported into EndNote, duplicates were removed, and then transferred to Covidence, a web-based software platform to streamline managing systematic reviews.^18^ All records were screened in duplicate. Review authors (NH-S and either YQ/MW) independently screened the title and abstract of each record against the eligibility criteria, then assessed the full-text articles for eligibility. Any uncertainty or disagreement was resolved through discussion and consensus. During the full-text review, reasons for exclusion were documented.

### Data extraction

All data were extracted in duplicate. Review authors (NH-S and one of TF/YL/TEL/HH) independently conducted data extraction using a customised extraction form on Covidence. Any discrepancies were resolved through discussion and by cross-checking the original studies. The following data were extracted from eligible studies: study design, country of origin, sample size, participant demographics (age, sex), SCI characteristics (time since injury, type of injury, type of paralysis, level of injury), pain measures and their score ranges, psychosocial outcomes and corresponding score ranges, and the direction and significance of correlations between pain intensity and psychosocial variables. Correlations were extracted from a single cross-sectional time point. For intervention-based studies, this corresponded to baseline (pre-intervention) assessments, whereas for longitudinal studies, correlations were extracted from the first time point at ≥1 year post-injury, to ensure that all included data reflected chronic SCI, as per the eligibility criteria. Only bivariate correlations were extracted. This focus on bivariate analyses was due to their prevalence in the literature, whereas the variability in multivariate models limited meaningful cross-study comparisons.

To ensure maximal coverage and data completeness, where studies assessed pain intensity and psychosocial outcomes without reporting correlations, NH-S contacted authors for up to three times to request raw data. If raw data were provided, NH-S calculated the correlation coefficients (*r*) in R (version 4.4.0)^19^ to assess the relationship between pain intensity and psychosocial outcome scores. A linear regression model was fitted, and the assumptions of normality and linearity were checked to confirm the correlation coefficients resulting from the calculations. If raw data were unavailable or not provided, the study was excluded.

### Quality assessment

Study quality was assessed in duplicate. Review authors (NH-S and one of TF/YL/TEL/HH/LA) independently assessed study quality, with discrepancies resolved via discussion. The methodological quality of included studies was evaluated using an adapted version of quality assessment tools developed by the US National Institutes of Health (NIH)^20^ and those employed in previous similar systematic reviews.^21^ The quality assessment tool used in this review included items assessing: study objective, population description, recruitment, response rate, sample size justification, pain characteristics, assessment methods and tools, and statistical analyses (eTable 1, Appendix 2). Each item of the quality assessment was rated as Yes (1), No (0), or Other (not applicable or cannot determine). A total quality score was calculated for each study, with ratings classified into three categories: low (<50%), medium (50%–79%), and high (≥80%) quality.

### Data analysis

Random-effects meta-analyses were conducted to examine the strength of association between pain intensity and each psychosocial factor using the *metafor* package (version 4.8.0) in R.^22^ Random-effects models were used to account for potential heterogeneity across studies. Meta-analyses were conducted only when bivariate correlation coefficients (*r*) were available from at least five studies, based on empirical evidence that random-effects meta-analyses with fewer than five studies often have lower statistical power than the individual studies themselves, leading to less reliable pooled estimates.^23^ Reported or calculated *r* values were first transformed to Fisher's *z* to stabilise variance and normalise the distribution.^24^ The variance of each transformed *r* was calculated as 1/(n−3), where n is the sample size of the study. The pooled *z* value was then back-transformed to *r* using the inverse Fisher's transformation for interpretability. Forest plots were generated to display the weighted effect size estimates from individual studies with 95% confidence intervals (CIs), alongside the pooled estimate and 95% CI. Studies with larger sample sizes contributed greater weight due to smaller variances and therefore had more influence on the pooled estimate.

Between-study heterogeneity was assessed using Cochran's Q-test, I^2^, and τ^2^ statistics.^24^^,^^25^ The Q-test evaluates whether the observed variability exceeds chance expectations, with a *p*-value of <0.1, indicating statistically significant heterogeneity. I^2^ quantifies the proportion of the observed variability which is due to between-study heterogeneity rather than sampling error.^26^ Cochrane guidelines suggest that I^2^ of 0–40% may be unimportant, 30–60% moderate, 50–90% substantial, and 75–100% considerable heterogeneity.^27^ Interpretation depends on the effect size magnitude and direction, and Q-test significance. τ^2^, estimated using the restricted maximum likelihood (REML), reflects absolute heterogeneity,^27^ with near-zero values indicating minimal variability, and larger values indicating greater dispersion in true effects across studies.^24^

For meta-analyses indicating significant between-study heterogeneity (i.e., a statistically significant Q-test), we conducted meta-regression analyses to examine whether the heterogeneity could be explained by study-level moderators,^24^^,^^27^ including sex distribution (proportion of female participants), mean age, mean time since injury, proportion with tetraplegia, proportion with complete injury, and the score ranges of pain and psychosocial scales. Each moderator was tested in a separate univariable meta-regression, except for the ranges of pain and psychosocial scales, which were examined together.

Post-hoc sensitivity analyses were conducted to assess the robustness of findings. For constructs with at least five eligible studies, separate analyses were performed excluding studies that (1) did not explicitly require chronic pain as an eligibility criterion or did not clearly report pain duration exceeding three months, (2) were rated as low quality, and (3) were intervention-based studies, to evaluate the potential influence of study design.

### Publication bias and certainty of evidence

Potential publication bias was examined visually using funnel plots and statistically using Egger's regression test and the trim-and-fill method for each meta-analysis. Funnel plot asymmetry was indicative of potential bias when Egger's test resulted in *p* < 0.05. For meta-analyses including <10 studies, publication bias assessment was not considered reliable. The trim-and-fill method was used to estimate the number of studies potentially missing and calculate adjusted pooled effect sizes.

Certainty of evidence was not formally assessed using the Grading of Recommendations, Assessment, Development and Evaluations (GRADE) as the majority of included studies were cross-sectional or observational. Accordingly, the overall certainty of evidence would start at ‘low’ by default, limiting the interpretability and added value of a formal GRADE assessment.

### Role of the funding source

This work was supported by the Craig H. Neilsen Foundation, the Rebecca L. Cooper Medical Research Foundation, and the NSW Health. The funders had no role in study design, data collection, data analysis, data interpretation, or writing of the report. The authors were not paid to write this article. The corresponding author had full access to all data in the study and had final responsibility for the decision to submit for publication.

## Results

**Fig. 1:**
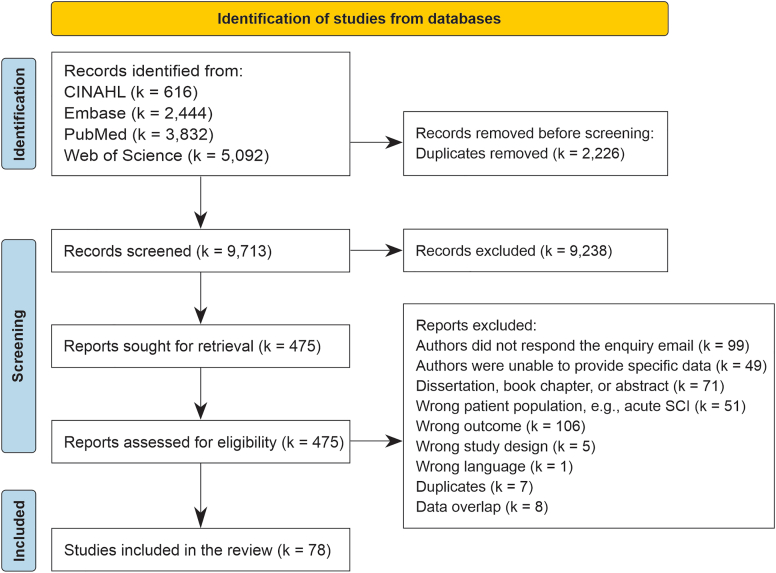
**PRISMA flow diagram.** Illustrating the selection process of studies from initial database searches to final inclusion in the systematic review.

The literature search is summarised in a PRISMA flow diagram (Fig. 1). Electronic database searches identified 11,939 potentially eligible articles. After de-duplication, title, abstract, and full-text screening, 78 studies were included in the review.

The included studies consisted of 48 cross-sectional, 15 longitudinal, eight randomised, three non-randomised, and four quasi-experimental studies. Sample sizes varied between nine^28^ and 4976^29^ participants. While six studies did not report the sex breakdown or did not specify it for the SCI subsample,^28^^,^^30^, ^31^, ^32^, ^33^, ^34^ two studies included only females,^35^^,^^36^ and the remaining studies reported male participation ranging from 44% to 100%. The mean age of participants ranged from 29.4^37^ to 59.6^38^ years. Mean time since injury varied extensively from one year^29^^,^^39^^,^^40^ to 25 years.^41^ Most studies were conducted in the United States (k = 36), Australia (k = 12), and the Netherlands (k = 7), with the remainder across 14 other countries (full details in eTable 3, Appendix 3).

Overall methodological quality was rated as moderate in the majority of studies (65 of 78), with seven studies rated as high and six as low quality (eTable 2, Appendix 2). Common methodological limitations included unclear reporting of response rates, lack of a priori sample size justification, and insufficient detail regarding medication use and pain characteristics. Measurement validity and reliability for pain and psychosocial outcomes were generally adequate, although variability in measurement tools limited direct comparability across studies.

Psychosocial outcomes were assessed using a range of validated self-report instruments across the included studies. Only measures representing conceptually comparable constructs (e.g., depression, anxiety, self-efficacy, catastrophising) were synthesised within each meta-analysis. Psychosocial constructs were defined based on established theoretical frameworks and grouped into several domains: mental health (e.g., depression, anxiety, psychological health); adaptive psychological factors (e.g., self-efficacy, acceptance, resilience); cognitive and emotional factors (e.g., catastrophising, stress, anger); social and interpersonal factors (e.g., social functioning, integration, and support); fatigue and sleep; and quality of life/life satisfaction. Table 1 summarises study characteristics across meta-analyses, including instruments used for each construct, brief conceptual definitions, study designs, quality ratings, and reporting of pain chronicity.

**Table 1:** Study characteristics across meta-analyses: pain and psychosocial measures, study design, study quality, and chronicity of pain.

Psychosocial measures	Construct definition	Pain measures	Study design	Study quality	Chronic pain?
**Mental health factors**					
**Depression (k = 49, n = 8185)** PHQ-9, PHQ-8, and PHQ-2 (k = 17) HADS-D (k = 11) CES-D (k = 8) PROMIS–Depression (k = 3) POMS–Depression (k = 3) BDI and BDI-II (k = 4) DASS-21–Depression (k = 2) HAM-D (k = 1)	Symptoms of low mood, loss of interest in activities, and related affective and cognitive disturbances.	NRS (k = 24) VAS (k = 4) BPI (k = 5) SF-36–bodily pain (k = 3) SF-MPQ (k = 2) MPI–pain severity (k = 3) ISCIPBDS–pain intensity (k = 2) 4-point scale (k = 2) PROMIS–pain intensity (k = 1) WBPQ (k = 1) CPG (k = 1) NPSI (k = 1)	*Observational:* Cross-sectional (k = 28) Longitudinal (k = 11) *Intervention-based:* Randomised controlled trial (k = 6) Non-randomised trial (k = 3) Quasi-experimental (k = 1)	High (k = 5) Medium (k = 42) Low (k = 2)	Yes (k = 14)
**Anxiety (k = 23, n = 2799)** HADS-A (k = 9) STAI-Trait (k = 3) GAD-7 (k = 3) PROMIS–Anxiety (k = 3) DASS-21–Anxiety (k = 2) POMS–Anxiety (k = 2) BAI (k = 1)	Excessive worry, tension, and physiological arousal reflecting heightened threat perception.	NRS (k = 14) BPI (k = 2) VAS (k = 1) SF-36–bodily pain (k = 1) SF-MPQ (k = 1) WBPQ (k = 1) ISCIPBDS–pain intensity (k = 1) PROMIS–pain intensity (k = 1) MPI (k = 1)	*Observational:* Cross-sectional (k = 14) Longitudinal (k = 7) *Intervention-based:* Randomised controlled trial (k = 2)	High (k = 3) Medium (k = 19) Low (k = 1)	Yes (k = 5)
**Psychological Health (k = 18, n = 2232)** WHOQOL-BREF–Psychological (k = 8) MHI-5 (k = 5) SF-36–Mental Health (k = 3) SF-12–Mental Component (k = 2)	Overall emotional well-being, including perceived mental functioning and absence of distress.	NRS (k = 13) VAS (k = 2) NPSI (k = 1) ISCIPEDS–pain intensity (k = 1) 6-point scale (k = 1)	*Observational:* Cross-sectional (k = 10) Longitudinal (k = 5) *Intervention-based:* Randomised controlled trial (k = 2) Quasi-experimental (k = 1)	High (k = 2) Medium (k = 15) Low (k = 1)	Yes (k = 5)
**Adaptive psychological factors**					
**Self-efficacy (k = 16, n = 2205)** MSES (k = 10) GSES (k = 4) PSEQ (k = 1) Self–efficacy Scale (Dutch) (k = 1)	Beliefs about one's capability to manage pain and its consequences.	NRS (k = 9) ISCIPBDS–pain intensity (k = 2) BPI (k = 2) 6-point scale (k = 1) PROMIS–pain intensity (k = 1) WBPQ (k = 1)	*Observational:* Cross-sectional (k = 10) Longitudinal (k = 4) *Intervention-based:* Randomised controlled trial (k = 2)	High (k = 3) Medium (k = 12) Low (k = 1)	Yes (k = 5)
**Acceptance (k = 9, n = 749)** ICQ–Acceptance (k = 4) SCL-CSQ–Acceptance (k = 2) CPAQ and CPAQ-8 (k = 2) AAQ (k = 1)	Willingness to experience pain without excessive attempts to control or avoid it, while engaging in valued activities.	NRS (k = 3) VAS (k = 2) BPI (k = 2) CPG (k = 1) 6-point scale (k = 1)	*Observational:* Cross-sectional (k = 6) Longitudinal (k = 1) *Intervention-based:* Randomised controlled trial (k = 2)	High (k = 2) Medium (k = 7)	Yes (k = 4)
**Resilience (k = 5, n = 5720)** CD-RISC-10 (k = 2) Brief Resilience Scale (k = 2) SCI-QOL–Resilience (k = 1)	Capacity to adapt positively to adversity and maintain or regain psychological functioning.	NRS (k = 2) BPI (k = 3)	*Observational:* Cross-sectional (k = 4) *Intervention-based:* Randomised controlled trial (k = 1)	Medium (k = 5)	Yes (k = 1)
**Cognitive and emotional factors**					
**Catastrophising (k = 18, n = 1324)** PCS (k = 7) CSQ–Catastrophising (k = 6) PRSS–Catastrophising (k = 3) PCL–Catastrophising (k = 1) PCCL–Catastrophising (k = 1)	Maladaptive cognitive response to pain characterised by rumination, magnification, and feelings of helplessness.	NRS (k = 10) BPI (k = 3) CPG (k = 2) SF-MPQ (k = 1) MPS–upper extremity (k = 1) WBPQ (k = 1)	*Observational:* Cross-sectional (k = 9) Longitudinal (k = 3) *Intervention-based:* Randomised controlled trial (k = 4) Non-randomised trial (k = 2)	High (k = 3) Medium (k = 14) Low (k = 1)	Yes (k = 9)
**Stress (k = 6, n = 437)** PSS and PSS-4 (k = 4) DASS-21–Stress (k = 2)	Perceived imbalance between environmental demands and an individual's coping resources.	NRS (k = 4) VAS (k = 1) MPI–pain severity (k = 1)	*Observational:* Cross-sectional (k = 4) Longitudinal (k = 1) *Intervention-based:* Non-randomised trial (k = 1)	Medium (k = 5) Low (k = 1)	Yes (k = 2)
**Anger (k = 5, n = 498)** POMS–Anger (k = 4) STAX–Trait Anger (k = 1)	Emotional response characterised by irritation or hostility, often linked to perceived injustice or frustration.	NRS (k = 1) SF-MPQ (k = 1) MPI–pain severity (k = 1) WHYMPI (k = 1) CPG (k = 1)	*Observational:* Cross-sectional (k = 5)	High (k = 1) Medium (k = 4)	Yes (k = 1)
**Social and interpersonal factors**					
**Social functioning (k = 10, n = 890)** WHOQOL-BREF–Social Relationships (k = 8) PROMIS–Satisfaction with Social Role (k = 1) SF-36–Social Functioning (k = 1)	Perceived ability to engage in and perform social roles and activities.	NRS (k = 7) VAS (k = 2) NPSI (k = 1)	*Observational:* Cross-sectional (k = 4) Longitudinal (k = 4) *Intervention-based:* Randomised controlled trial (k = 2)	High (k = 2) Medium (k = 8)	Yes (k = 1)
**Social integration (k = 7, n = 3353)** CHART–Social Integration (k = 3) CIQ–Social Integration (k = 3) Community Activities–Social Interaction (k = 1)	Objective participation in social activities and involvement in community and interpersonal networks.	NRS (k = 4) VAS (k = 1) 4-point scale (k = 1) MPI–pain severity (k = 1)	*Observational:* Cross-sectional (k = 6) Longitudinal (k = 1)	High (k = 1) Medium (k = 4) Low (k = 2)	Yes (k = 1)
**Social support (k = 6, n = 1631)** MSPSS (k = 2) SSQ-6 (k = 1) SSL-12 (k = 1) SHP–social support (k = 1) MPI–social support (k = 1)	Perceived or received emotional, informational, or practical assistance from others.	NRS (k = 3) PROMIS–pain intensity (k = 1) ISCIPBDS–pain intensity (k = 1) MPI–pain severity (k = 1)	*Observational:* Cross-sectional (k = 4) Longitudinal (k = 1) *Intervention-based:* Quasi-experimental (k = 1)	High (k = 1) Medium (k = 4) Low (k = 1)	Yes (k = 2)
**Fatigue and Sleep**					
**Fatigue (k = 10, n = 1614)** FSS (k = 4) POMS–Fatigue (k = 3) PROMIS–Fatigue (k = 2) NRS (k = 1)	Subjective experience of persistent physical or mental exhaustion that interferes with functioning.	NRS (k = 6) BPI (k = 1) 4-point scale (k = 1) PROMIS–pain intensity (k = 1) SF-MPQ (k = 1)	*Observational:* Cross-sectional (k = 6) Longitudinal (k = 4)	High (k = 1) Medium (k = 8) Low (k = 1)	Yes (k = 1)
**Sleep (k = 9, n = 1888)** PROMIS–Sleep Disturbance (k = 3) PSQI (k = 3) BNSQ (k = 2) NRS (k = 1)	Perceived difficulties in sleep continuity or adequacy, including non-restorative or insufficient sleep.	NRS (k = 3) BPI (k = 2) VAS (k = 1) NPSI (k = 1) PROMIS–pain intensity (k = 1) MPI–pain severity (k = 1)	*Observational:* Cross-sectional (k = 4) Longitudinal (k = 3) *Intervention-based:* Randomised controlled trial (k = 1) Quasi-experimental (k = 1)	Medium (k = 8) Low (k = 1)	Yes (k = 2)
**Quality of life and life satisfaction**					
**Quality of life/life satisfaction (k = 16, n = 3722)** SWLS (k = 8) WHOQOL-BREF–QoL (k = 3) SQoL (k = 2) LSI-A (k = 1) M-HIP–Life Satisfaction item (k = 1) SV-QLI/SCI (k = 1)	Overall perception of health-related quality of life and satisfaction with life.	NRS (k = 6) VAS (k = 3) ISCIPBDS–pain intensity (k = 1) SF-36–bodily pain (k = 1) WBPQ (k = 1) PROMIS–pain intensity (k = 1) M-HIP (k = 1) MPS–upper extremity (k = 1) 5-point scale (k = 1)	*Observational:* Cross-sectional (k = 5) Longitudinal (k = 4) *Intervention-based:* Randomised controlled trial (k = 4) Non-randomised trial (k = 2) Quasi-experimental (k = 1)	High (k = 4) Medium (k = 8) Low (k = 4)	Yes (k = 3)

**AAQ:** Acceptance and Action Questionnaire; **BAI:** Beck Anxiety Inventory; **BDI:** Beck Depression Inventory; **BNSQ:** Basic Nordic Sleep Questionnaire; **BPI:** Brief Pain Inventory; **CD-RISC-10:** Connor-Davidson Resilience Scale; **CES-D:** Centre for Epidemiological Studies–Depression; **CHART:** Craig Handicap Assessment and Reporting Technique; **CIQ:** Community Integration Questionnaire; **CPAQ:** Chronic Pain Acceptance Questionnaire; **CPG:** Chronic Pain Grade scale; **CSQ:** Coping Strategies Questionnaire; **DASS-21:** Depression Anxiety Stress Scale; **FSS:** Fatigue Severity Scale; **GAD-7:** Generalized anxiety disorder scale-7; **GSES:** General Self–Efficacy Scale; **HADS:** Hospital Anxiety and Depression Scale; **HAM-D:** Hamilton Rating Scale for Depression; **ICQ:** Illness Cognitions Questionnaire; **ISCIPBDS:** International Spinal Cord Injury Pain Basic Data Set; **LSI-A:** Life Satisfaction Index-A; **MHI-5:** Mental Health Index-5; **M-HIP:** McGuire Health Impact on Participation Survey; **MPI:** Multidimensional Pain lnventory; **MPS:** Musculoskeletal Pain Survey; **MSES:** Moorong Self–Efficacy Scale; **MSPSS:** Multidimensional Scale of Perceived Social Support; **NPSI:** Neuropathic Pain Symptom Inventory; **NRS:** Numerical Rating Scale; **PCCL:** Pain Coping and Cognition List; **PCL:** Pain Cognition List-2003; **PCS:** Pain Catastrophizing Scale; **PHQ:** Patient Health Questionnaire; **POMS:** Profile of Mood States; **PROMIS:** Patient-Reported Outcomes Measurement Information System; **PRSS:** Pain-Related Self–Statement Scale; **PSEQ:** Pain Self-Efficacy Questionnaire; **PSQI:** Pittsburgh Sleep Quality Index; **PSS:** Perceived stress scale; **SCI-QOL:** Spinal Cord Injury-Quality of Life; **SCL-CSQ:** Spinal cord lesion-related coping strategy questionnaire; **SHP:** Swiss Household Panel; **SF:** Short Form Health Survey; **SFMPQ:** Short-Form McGill Pain Questionnaire; **SQoL:** Subjective Quality of Life; **SSL-12:** Social Support List-12; **SSQ-6:** Social Support Questionnaire-6; **STAI:** State-Trait Anxiety Index; **STAX:** State-Trait Anger Expression Inventory; **SWLS:** Satisfaction with Life Scale; **SV-QLI/SCI:** Subjective Vancouver-Quality of Life Index for Spinal Cord Injury; **VAS:** Visual Analogue Scale; **WBPQ:** Wisconsin brief pain questionnaire; **WHOQOL-BREF:** World Health Organization Quality of Life–brief version; **WHYMPI:** West Haven–Yale Multidimensional Pain Inventory.

Across domains, higher pain intensity was consistently associated with poorer psychosocial functioning, although the magnitude and consistency of associations varied by construct. Pooled estimates for each meta-analysis are presented below, alongside results from meta-regression analyses. A summary of meta-regression findings, indicating how each tested moderator influenced the associations, is provided in eTable 4, Appendix 4.

**Fig. 2:**
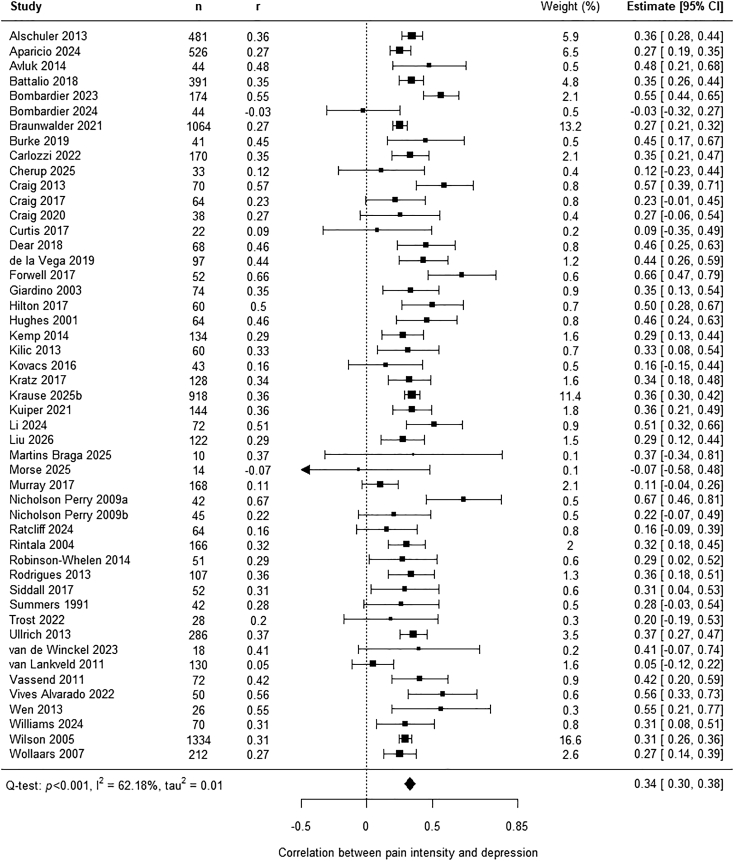
**Forest plot of random-effects model meta-analysis for the pain–depression association across 49 studies.** Individual study sample sizes, correlation coefficients (*r*), and 95% confidence intervals (CIs) are shown. Square sizes are proportional to study weights, and the diamond represents the pooled effect size estimate (*r* = 0.34, 95% CI [0.30, 0.38]). Positive correlations indicate that higher pain is associated with more severe depression symptoms. The plot is displayed on the *r* scale (back-transformed from Fisher's *z*), which resulted in wider spacing near the extremes.

Mental health factors were examined across several constructs. *Depression* was assessed in 49 studies (n = 8185). Higher pain intensity was moderately associated with more severe depressive symptoms (*r* 0.34, 95% CI 0.30 to 0.38; *p* < 0.0001; Fig. 2). Heterogeneity was substantial (Q(48) 103.78; *p* < 0.0001; I^2^ 62.18%; τ^2^ 0.01), but none of the tested moderators significantly explained this heterogeneity.

**Fig. 3:**
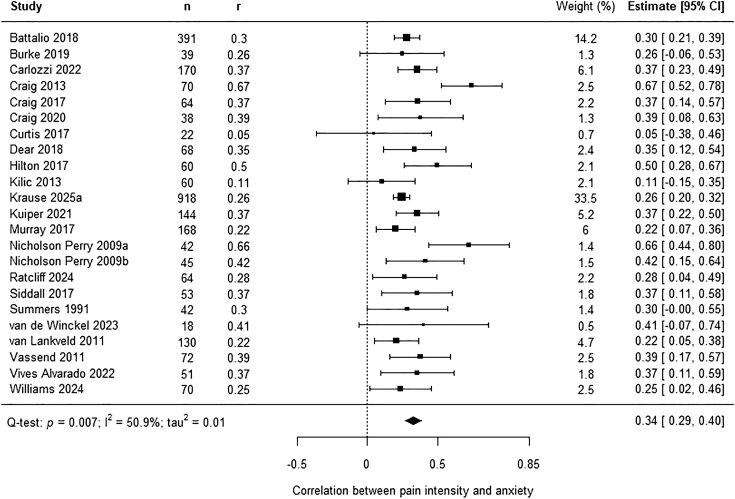
**Forest plot of pain–anxiety association across 23 studies**.

*Anxiety* was evaluated in 23 studies (n = 2799). Higher pain intensity was moderately associated with more severe levels of anxiety (*r* 0.34, 95% CI 0.28 to 0.40; *p* < 0.0001; Fig. 3), with moderate heterogeneity (Q(22) 41.52; *p* = 0.007; I^2^ 50.90%; τ^2^ 0.01). Meta-regression identified the female proportion as a significant moderator, explaining 47% of heterogeneity (QM(1) 5.50; *p* = 0.019; R^2^ 47.55%). This association became weaker as the proportion of female participants increased, indicating a stronger pain–anxiety relationship in studies with more male participants. Other moderators did not reach statistical significance.

*Psychological health* was assessed in 18 studies (n = 2232). Higher pain intensity was moderately associated with lower psychological health (*r* −0.30, 95% CI −0.36 to −0.24; *p* < 0.0001; eFig. 1, Appendix 4), with moderate heterogeneity (Q(17) 28.46, *p* = 0.040; I^2^ 37.64%; τ^2^ 0.01). Meta-regression suggested that age and the proportion of participants with tetraplegia may explain a percentage of between-study heterogeneity (R^2^ 60.48% and 38.41%, respectively). However, neither moderator reached statistical significance (age QM(1) 3.07; *p* = 0.080; tetraplegia QM(1) 1.83; *p* = 0.176), indicating uncertainty in these estimates. No other moderators were significant.

**Fig. 4:**
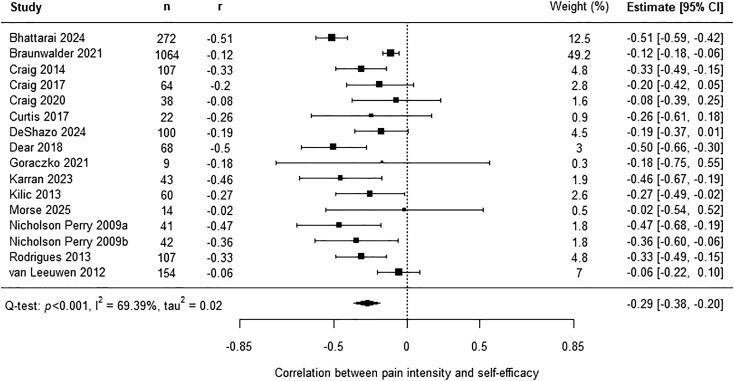
**Forest plot of association between pain and self-efficacy across 16 studies**.

Adaptive psychological factors were assessed across self-efficacy, acceptance, and resilience. *Self-efficacy* was reported in 16 studies (n = 2205). Pain intensity was moderately associated with lower self-efficacy (*r* −0.29, 95% CI −0.38 to −0.20; *p* < 0.0001; Fig. 4), with substantial heterogeneity (Q(15) 64.38; *p* < 0.0001; I^2^ 69.39%; τ^2^ 0.02). Age accounted for 41% of heterogeneity (QM(1) 5.54; *p* = 0.019; R^2^ 41.38%), and the association weakened with older age, indicating a stronger association in younger adults. A separate model including pain and self–efficacy scale ranges explained 39% of heterogeneity (QM(2) 4.78; *p* = 0.091; R^2^ 39.67%) but did not reach statistical significance, indicating uncertainty in the estimate. All other moderators were non-significant and explained little or no additional variability.

*Acceptance* was examined in nine studies (n = 749). Pain intensity was moderately associated with lower levels of acceptance (*r* −0.27, 95% CI −0.38 to −0.16; *p* < 0.0001; eFig. 2, Appendix 4), with moderate heterogeneity (Q(8) 19.27; *p* = 0.013; I^2^ 56.86%; τ^2^ 0.02). Time since injury significantly moderated the association (QM(1) 7.15; *p* = 0.008), fully accounting for heterogeneity (R^2^ 100%). Longer time since injury strengthened the association. No other moderators reached statistical significance.

*Resilience* was assessed in five studies (n = 5720). Higher pain intensity was moderately associated with lower resilience (*r* −0.27, 95% CI −0.29 to −0.24; *p* < 0.0001; eFig. 3, Appendix 4). No significant heterogeneity was observed (Q(4) 7.55; *p* = 0.11; I^2^ 0.07%; τ^2^ 0.00), and no meta-regression was conducted.

**Fig. 5:**
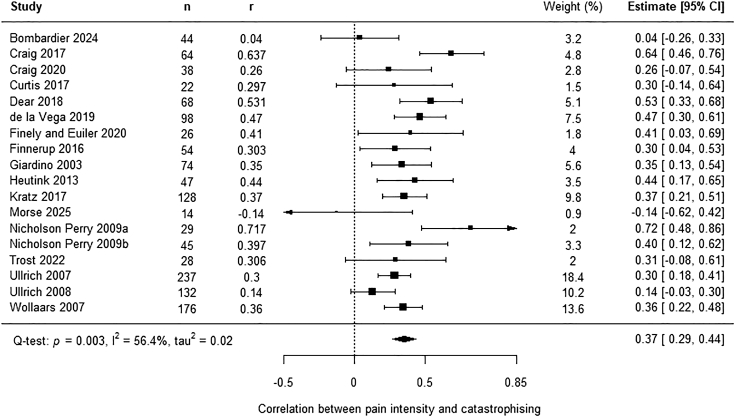
**Forest plot of pain–catastrophising association across 18 studies**.

Cognitive and emotional factors were examined across catastrophising, stress, and anger. *Catastrophising* was reported in 18 studies (n = 1324). Pain intensity was moderately associated with higher catastrophising (*r* 0.37, 95% CI 0.29 to 0.44; *p* < 0.0001; Fig. 5), with substantial heterogeneity (Q(16) 37.71; *p* = 0.003; I^2^ 56.40%; τ^2^ 0.02). Time since injury significantly moderated the association (QM(1) 11.80; *p* < 0.001), explaining 96% of heterogeneity (R^2^ 96.26%). Longer time since injury weakened the correlation, suggesting a stronger association earlier after injury. Additional meta-regression models suggested that age and the proportion of female participants may explain a percentage of between-study heterogeneity (R^2^ 22.65% and 23.38%, respectively). However, neither moderator reached statistical significance (age QM(1) 3.58; *p* = 0.058; female percentage QM(1) 1.88; *p* = 0.170), indicating uncertainty in these estimates. No other moderators explained additional variability.

*Stress* was assessed in six studies (n = 437). Higher pain intensity was weakly associated with higher levels of stress (*r* 0.19, 95% CI 0.10 to 0.28; *p* < 0.0001; eFig. 4, Appendix 4). No significant heterogeneity was observed (Q(5) 5.22; *p* = 0.39; I^2^ 0.04%; τ^2^ 0.00), and no meta-regression was conducted.

*Anger* was assessed in five studies (n = 498). Pain intensity was moderately associated with higher levels of anger (*r* 0.34, 95% CI 0.17 to 0.49; *p* = 0.0001; eFig. 5, Appendix 4), with substantial heterogeneity (Q(4) 14.09, *p* = 0.007; I^2^ 73.45%; τ^2^ 0.03). The proportion of participants with tetraplegia significantly moderated the association (QM(1) 7.18; *p* = 0.007), explaining 89% of heterogeneity (R^2^ 89.04%). Studies including more participants with tetraplegia showed a weaker association. Pain and anger scale ranges jointly moderated the association, accounting for 85% of heterogeneity (QM(2) 8.42; *p* = 0.015; R^2^ 85.21%), with wider ranges linked to smaller effect sizes. Female proportion was also a significant moderator, explaining 68% of heterogeneity (QM(1) 4.59; *p* = 0.032; R^2^ 68.31%). The association was weaker in samples with more females, suggesting a stronger association in studies with more male participants. Other moderators were non-significant.

**Fig. 6:**
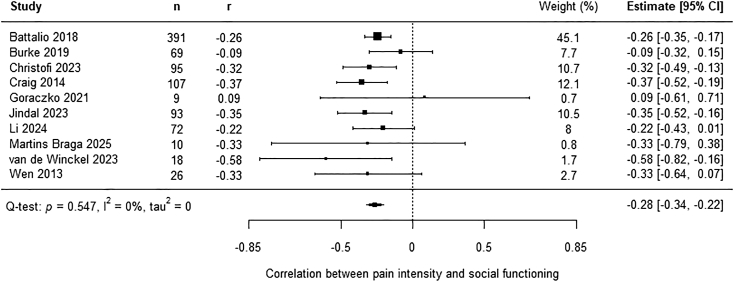
**Forest plot of pain and social functioning association across 10 studies**.

Social and interpersonal factors were assessed across several constructs. *Social functioning* was reported in ten studies (n = 890). Higher pain intensity was moderately associated with lower self-perceived social functioning (*r* −0.28, 95% CI −0.34 to −0.22; *p* < 0.0001; Fig. 6). No heterogeneity was observed (Q(9) 7.87; *p* = 0.55; I^2^ 0%; τ^2^ 0.00), and no meta-regression was conducted.

*Social integration* was examined in seven studies (n = 3353). Higher pain intensity was weakly associated with lower social integration (*r* −0.14, 95% CI −0.24 to −0.04; *p* = 0.006; eFig. 6, Appendix 4). Heterogeneity was substantial (Q(6) 14.15; *p* = 0.028; I^2^ 66.55%; τ^2^ 0.01), but all tested moderators were non-significant.

*Social support* was assessed across six studies (n = 1631). Pain intensity was not significantly associated with social support (*r* −0.05, 95% CI −0.28 to 0.20; *p* = 0.72; eFig. 7, Appendix 4), and heterogeneity was considerable (Q(5) 57.95; *p* < 0.0001; I^2^ 93.37%; τ^2^ 0.08). Meta-regression indicated that the proportion of participants with tetraplegia significantly moderated the association (QM(1) 6.76, *p* = 0.009), explaining nearly all between-study heterogeneity (R^2^ 99.99%). As the proportion of participants with tetraplegia increased, the association became less negative, indicating a weaker pain–social support relationship in studies with a higher proportion of tetraplegia. The proportion of female participants was also a significant moderator (QM(1) 4.79; *p* = 0.029; R^2^ 55.54%), with stronger negative associations observed in studies with higher female representation. A separate model suggested that the proportion of participants with complete injury may explain some heterogeneity (R^2^ 37.62%), but this did not reach statistical significance (QM(1) 9.31; *p* = 0.052). Other moderators were non-significant and explained little or no additional variance.

**Fig. 7:**
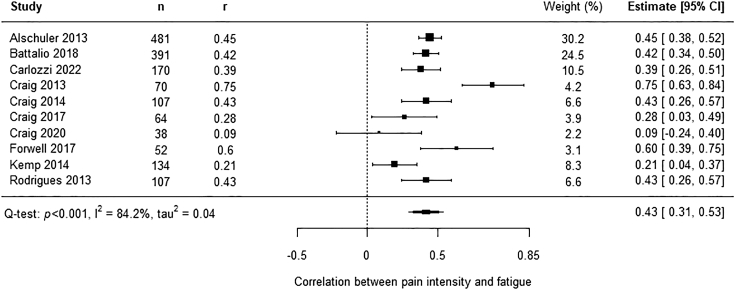
**Forest plot of pain–fatigue association across 10 studies**.

Fatigue and sleep outcomes were examined across relevant measures. *Fatigue* was assessed in 10 studies (n = 1614). Higher pain intensity was moderately associated with greater fatigue (*r* 0.43, 95% CI 0.31 to 0.53; *p* < 0.0001; Fig. 7), with considerable heterogeneity (Q(9) 35.52; *p* < 0.0001; I^2^ 84.20%; τ^2^ 0.04). Pain scale range significantly moderated the association, with wider scales showing stronger correlations, whereas fatigue scale range and other moderators were not significant. Other moderators did not reach statistical significance.

*Sleep* disturbance was measured in 9 studies (n = 1888). Higher pain intensity was weakly associated with sleep disturbance (*r* 0.23, 95% CI 0.00 to 0.44; *p* = 0.048; eFig. 8, Appendix 4), and heterogeneity was considerable (Q(8) 86.03; *p* < 0.0001; I^2^ 94.15%; τ^2^ 0.10). All tested moderators were non-significant and explained little or no variability.

**Fig. 8:**
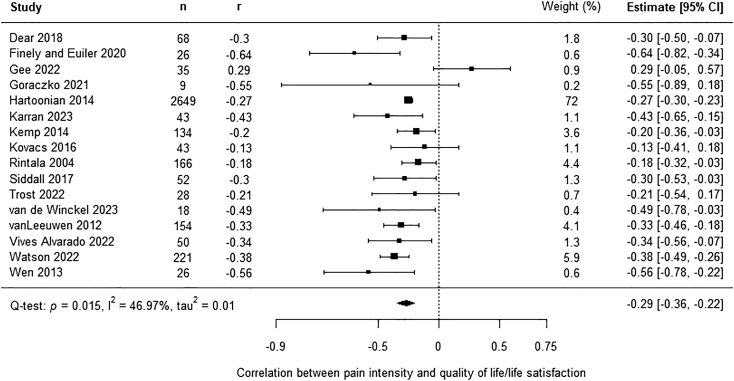
**Forest plot of association between pain and quality of life/life satisfaction across 16 studies**.

*Quality of life and life satisfaction* were assessed in 16 studies (n = 3722). Higher pain intensity was moderately associated with lower quality of life and life satisfaction (*r* −0.29, 95% CI −0.36 to −0.22; *p* < 0.0001; Fig. 8), with moderate heterogeneity (Q(15) 29.33; *p* = 0.015; I^2^ 46.97%; τ^2^ 0.01). Meta-regression indicated that the proportion of participants with tetraplegia significantly moderated the association (QM(1) 3.88; *p* = 0.049), explaining only 17% of between-study heterogeneity (R^2^ 17.08%). Other tested moderators were non-significant and explained little or no additional variability.

Sensitivity analyses excluding studies with unclear pain chronicity resulted in consistent estimates, with two exceptions. Stronger association was observed for pain–self-efficacy, and weaker association for pain and psychological health; in both cases, heterogeneity was reduced to non-significant (eTable 5, Appendix 5). Excluding low-quality studies indicated similar findings, with the exception of the pain–social integration association, which became non-significant, accompanied by increased heterogeneity (eTable 6, Appendix 5). Further, excluding intervention-based studies (e.g., randomised or non-randomised trials) produced results largely consistent with primary findings. The only exceptions were the pain–sleep association, which became non-significant, and the pain–social support association, which increased in magnitude but remained non-significant. Heterogeneity remained considerable in both cases (eTable 7, Appendix 5). These changes in pooled estimates likely reflect differences in study composition and reduced sample sizes across sensitivity analyses, which can influence effect estimates and statistical significance, even when heterogeneity remains similar.

Funnel plots (eFig. 9–23, Appendix 6) were generally symmetrical, except for the pain–anger association, for which Egger's test suggested potential asymmetry (z 3.56; *p* < 0.001). Publication bias was further assessed using trim-and-fill analyses, which imputed a small number of studies in several meta-analyses, including catastrophising (k = 3), stress (k = 2), social support (k = 2), sleep (k = 2), and quality of life/life satisfaction (k = 3), as well as single studies for self-efficacy, resilience, and social functioning. However, adjusted pooled estimates were largely unchanged, with only minor shifts, and no meaningful impact on the overall interpretation of findings (full details in eTable 8, Appendix 6). The only exception was the pain–sleep association, where the adjusted estimate became smaller and no longer statistically significant, indicating less stable evidence.

## Discussion

This systematic review and meta-analysis synthesised 78 studies (n = 19,785 participants), providing a large evidence base for examining associations between pain intensity and psychosocial factors in individuals with SCI. Across domains, higher pain intensity was consistently associated with poorer psychosocial outcomes. Moderate correlations were observed with depression, anxiety, psychological health, self-efficacy, acceptance, resilience, catastrophising, anger, fatigue, and quality of life/life satisfaction. Associations with social variables were more variable, with moderate effect for social functioning, small effect for social integration, and non-significant for social support. Associations with stress and sleep disturbance were also weak. These findings extend prior work by quantifying the strength of these associations across a broader range of psychosocial factors than previously examined.

Previous studies demonstrated relationships between higher pain intensity and depression,^14^ anxiety,^14^ catastrophising,^42^^,^^43^ fatigue,^44^ stress,^14^ sleep disturbance,^45^^,^^46^ lower self-efficacy,^14^^,^^47^^,^^48^ lower acceptance,^14^ and reduced quality of life.^14^ Although catastrophising and coping are recognised predictors of pain-related outcomes in individuals with physical disability, including SCI,^49^ relatively few included studies assessed coping, highlighting a gap in the literature. The non-significant association with social support contrasts with reports of lower support among people with more severe pain,^50^ potentially reflecting differences in sample or measurement tools.

The substantial heterogeneity observed across many analyses likely reflects methodological differences between studies, including variability in individual characteristics, differences in measurement tools, and variation in how psychosocial constructs and pain outcomes were defined and measured. Although random-effects models and moderator analyses were used to explore some of the potential sources of variability, these differences should be considered when interpreting pooled estimates.

Although publication bias was assessed using standard methods, the possibility that studies reporting non-significant associations remain unpublished cannot be fully excluded. If present, such bias could lead to overestimation of the magnitude of some pooled associations.

Meta-regression analyses identified sex as a significant moderator for three of pain–psychosocial associations. Studies with more men showed stronger pain associations with anxiety and anger, whereas studies with more women showed stronger associations with lower social support. These patterns align with findings from non-SCI chronic pain populations, where anxiety predicts pain outcomes more strongly in men than women.^51^^,^^52^ Stronger pain–anger relationship in male-dominant samples may reflect patterns of emotional expression often observed in men, who are more likely to externalise anger.^53^ Stronger pain–social support associations in samples with more women may partly be explained by women's greater reliance on interpersonal coping strategies, as suggested in prior work on chronic pain.^53^^,^^54^ While some studies support sex-specific mechanisms, others report no moderation effects^55^ or highlight contextual complexities, such as cultural or psychosocial norms.^56^

Older age moderated only one association, with a weaker negative relationship with self-efficacy. These patterns are consistent with evidence from non-SCI chronic pain populations, where older adults report higher self-efficacy despite similar pain levels,^57^ and where the pain–self-efficacy relationship diminishes with age but remains unaffected by sex.^58^

Time since injury was a significant moderator for acceptance and catastrophising. Longer duration post-injury was associated with stronger pain associations with acceptance, and a weaker association with catastrophising, suggesting that psychosocial responses to pain evolve as individuals adjust to their injury. Acceptance may strengthen over time,^59^ whereas catastrophising may diminish with improved coping and emotion regulation.^60^ These findings are consistent with Craig's SCI adjustment model,^12^ which conceptualises psychosocial factors, such as catastrophising, as dynamic processes across the post-injury lifespan.

An existing systematic review^61^ reported no association between injury characteristics (including completeness and tetraplegia) and pain prevalence in SCI. In the current review, completeness of injury did not moderate any associations. However, the proportion of participants with tetraplegia influenced three associations, with higher proportions associated with weaker relationships between pain and anger, social support, and quality of life/life satisfaction. This pattern suggests that while injury characteristics may not influence the presence of pain, they may shape how pain relates to psychosocial outcomes, potentially reflecting the broader impact of higher-level injuries on daily life.^62^

Several limitations should be considered when interpreting these findings. First, this review synthesised cross-sectional associations, which limits causal inference regarding the directionality of the observed relationships. As most included studies were observational and cross-sectional, it is not possible to determine whether psychosocial factors contribute to greater pain intensity or whether persistent pain leads to changes in psychosocial functioning, and bidirectional relationships are likely. Second, several studies provided limited reporting of methodological and clinical characteristics, including sample size justification, participant characteristics (particularly medication use or engagement in therapies), and detailed pain characteristics such as duration or location. These reporting gaps may limit the ability to fully contextualise study findings and assess potential confounding factors (e.g., medication use or ongoing therapies), which may affect the interpretation, robustness, and generalisability of the pooled estimates. Third, most studies did not distinguish neuropathic from non-neuropathic pain, limiting the specificity of the findings. Fourth, although the review focused on chronic pain, many studies did not report pain duration. To address this, we included studies involving individuals with chronic SCI (≥1 year post-injury) and conducted sensitivity analyses restricted to studies explicitly reporting chronic pain, which produced consistent results. Fifth, psychosocial constructs were assessed using a wide range of measurement tools, which may have contributed to heterogeneity despite accounting for scale range in moderator analyses. Finally, because the evidence base was largely observational and cross-sectional, we did not apply GRADE, as the certainty of evidence would likely be rated as low and would provide limited additional interpretive value.

The findings of this systematic review and meta-analysis have several important clinical and theoretical implications. Pain following SCI reflects the interaction of biological, psychological, and social processes that evolve over time.^63^ The associations identified in this review support a biopsychosocial model^64^ of SCI-related pain. These findings align with multilevel frameworks of adjustment after SCI, such as Craig's dynamic model,^12^ which emphasises the evolving interplay between biological injury, psychological adaptation, and social context across the post-injury lifespan.

From a clinical perspective, these findings highlight potential targets for psychosocially informed pain management. Interventions addressing maladaptive psychological, cognitive, and emotional factors may complement biomedical approaches. While the evidence for the efficacy of psychological interventions, such as cognitive behavioural therapies, remains uncertain for pain intensity as an outcome, these approaches have shown promise in improving comorbid mental health problems in people with SCI-related pain^6^ or in non-SCI chronic pain population.^65^ Integrating such approaches within multidisciplinary neurorehabilitation frameworks may support more holistic and personalised pain management strategies.

Given the cross-sectional nature of the included evidence, future research should prioritise longitudinal and intervention-based designs to clarify temporal dynamics and causal pathways. Complementary syntheses focusing on longitudinal processes and treatment effects may further advance understanding of underlying mechanisms and inform the development of targeted psychosocial interventions within integrated rehabilitation pathways.

In conclusion, this systematic review provides the first comprehensive synthesis of associations between pain intensity and a wide range of psychosocial factors in chronic SCI. Findings highlight the importance of a biopsychosocial approach to SCI pain management, and point to the moderation roles of sex, time since injury, and injury level, supporting the value of personalised approaches. Overall, this evidence base highlights the need to advance both mechanistic understanding and intervention development within neurorehabilitation frameworks.

## Contributors

All authors read and approved the final version of the manuscript. NH-S and YQ verified the underlying data. Author contributions are detailed below.

NH-S: conceptualisation, data curation, formal analysis, funding acquisition, investigation, methodology, project administration, software, validation, visualisation, writing–original draft.

YQ: conceptualisation, methodology, investigation, validation, writing–review & editing.

TEL: investigation, validation, writing–review & editing.

MAW: methodology, investigation, validation, writing–review & editing.

HJH: investigation, validation, writing–review & editing.

TFC: investigation, project administration, writing–review & editing.

YL: investigation, validation, writing–review & editing.

LA: investigation, validation, writing–review & editing.

TN-J: conceptualisation, funding acquisition, writing–review & editing.

JHM: conceptualisation, funding acquisition, writing–review & editing.

ZT: conceptualisation, funding acquisition, writing–review & editing.

SMG: conceptualisation, funding acquisition, supervision, writing–review & editing.

## Data sharing statement

The data and analysis code used in this study are publicly available on the Open Science Framework (https://osf.io/mqx7z/).

## Declaration of interests

Dr Yann Quidé is supported by a research grant from the Network of European Funding for Neuroscience Research (ERA-NET NEURON; JTC2025) and the Australian National Health and Medical Research Council (NHMRC; 2050486). The other authors declare no competing interests.

## Appendix A Supplementary data

Appendix 1 - Search Strategy

Appendix 2 - Study Quality

Appendix 3 - Study Characteristics

Appendix 4 - Statistical Analysis

Appendix 5 - Sensitivity Analysis

Appendix 6 - Publication Bias
