# Supplementary material for: Psychosocial factors associated with pain in spinal cord injury: a systematic review and meta-analysis
Source: eClinicalMedicine. 2026 May 18;96:103976. doi: 10.1016/j.eclinm.2026.103976 (PMC13316355; doi:10.1016/j.eclinm.2026.103976)
Supplement: Appendix 1 - Search Strategy [file mmc1.docx]

**Appendix 1 – Search Strategy**

Search updated in March 2026, to identify peer-reviewed studies, conducted on humans and published in English, with no restrictions on the publication year.

***PubMed***

#1 “spinal cord injury”[MeSH]

#2 ((“spinal cord”[tiab] OR spinal[tiab] OR spine[tiab] OR cervical[tiab]) AND (injur*[tiab] OR lesion*[tiab] OR trauma*[tiab] OR transection*[tiab] OR laceration*[tiab] OR damage*[tiab] OR fracture*[tiab] OR contusion*[tiab]))

#3 “transverse lesion”[tiab] OR “transverse cord lesion”[tiab] OR “traumatic myelopathy”[tiab] OR paraplegi*[tiab] OR quadriplegi*[tiab] OR tetraplegi*[tiab] OR SCI[tiab]

#4 #1 OR #2 OR #3

#5 pain[MeSH]

#6 ((chronic*[tiab] OR back[tiab] OR musculoskel*[tiab] OR muscle[tiab] OR nocicepti*[tiab] OR intractabl*[tiab] OR neuropath*[tiab] OR nerve[tiab] OR neck[tiab] OR central[tiab] OR abdominal[tiab]) AND pain*[tiab])

#7 #5 OR #6

#8 psychosocial[MeSH]

#9 psychosocial[tiab] OR psycholog*[tiab] OR mood[tiab] OR sleep[tiab] OR “mental disorder”[tiab] OR “mental illness”[tiab] OR depress*[tiab] OR anxiety[tiab] OR stress[tiab] OR posttrauma*[tiab] OR post-trauma*[tiab] OR anger[tiab] OR fatigue[tiab]

#10 “social support”[tiab] OR catastrophi*[tiab] OR cogniti*[tiab] OR coping[tiab] OR cope[tiab] OR accept*[tiab] OR satisfaction[tiab] OR belief*[tiab] OR self-efficacy[tiab] OR resilience[tiab]

#11 #8 OR #9 OR #10

#12 #4 AND #7 AND #11

***Web of Science***

#1 TS=(spinal cord injury)

#2 TI=((“spinal cord” OR spinal OR spine OR cervical) AND (injur* OR lesion* OR trauma* OR transection* OR laceration* OR damage* OR fracture* OR contusion*)) OR AB=((“spinal cord” OR spinal OR spine OR cervical) AND (injur* OR lesion* OR trauma* OR transection* OR laceration* OR damage* OR fracture* OR contusion*))

#3 TI=(“transverse lesion” OR “transverse cord lesion” OR “traumatic myelopathy” OR paraplegi* OR quadriplegi* OR tetraplegi* OR SCI) OR AB=(“transverse lesion” OR “transverse cord lesion” OR “traumatic myelopathy” OR paraplegi* OR quadriplegi* OR tetraplegi* OR SCI)

#4 #1 OR #2 OR #3

#5 TS=(pain)

#6 TI=((chronic* OR back OR musculoskel* OR muscle OR nocicepti* OR intractabl* OR neuropath* OR nerve OR neck OR central OR abdominal) AND pain*) OR AB=((chronic* OR back OR musculoskel* OR muscle OR nocicepti* OR intractabl* OR neuropath* OR nerve OR neck OR central OR abdominal) AND pain*)

#7 #5 OR #6

#8 TS=(psychosocial)

#9 TI=(psychosocial OR psycholog* OR mood OR sleep OR “mental disorder” OR “mental illness” OR depress* OR anxiety OR stress OR distress OR posttrauma* OR post-trauma* OR anger OR fatigue) OR AB=(psychosocial OR psycholog* OR mood OR sleep OR “mental disorder” OR “mental illness” OR depress* OR anxiety OR stress OR distress OR posttrauma* OR post-trauma* OR anger OR fatigue)

#10 TI=(“social support” OR catastrophi* OR cogniti* OR coping OR cope OR accept* OR satisfaction OR self-efficacy OR resilience) OR AB=(“social support” OR catastrophi* OR cogniti* OR coping OR cope OR accept* OR satisfaction OR self-efficacy OR resilience)

#11 #8 OR #9 OR #10

#12 #4 AND #7 AND #11 NOT TS=(rat OR rats OR mouse OR mice OR murine OR feline OR cat OR canine OR dog OR monkey OR primate OR "animal model*" OR "preclinical model*" OR sheep OR pig OR rabbit OR rodent OR "animal experiment*")

***CINAHL (via EBSCOhost)***

S1 MH “spinal cord injury”

S2 TI ((“spinal cord” OR spinal OR spine OR cervical) N4 (injur* OR lesion* OR trauma* OR transection* OR laceration* OR damage* OR fracture* OR contusion*)) OR AB ((“spinal cord” OR spinal OR spine OR cervical) N4 (injur* OR lesion* OR trauma* OR transection* OR laceration* OR damage* OR fracture* OR contusion*))

S3 TI (“transverse lesion” OR “transverse cord lesion” OR “traumatic myelopathy” OR paraplegi* OR quadriplegi* OR tetraplegi* OR SCI) OR AB (“transverse lesion” OR “transverse cord lesion” OR “traumatic myelopathy” OR paraplegi* OR quadriplegi* OR tetraplegi* OR SCI)

S4 S1 OR S2 OR S3

S5 MH “pain”

S6 TI ((chronic* OR back OR musculoskel* OR muscle OR nocicepti* OR intractabl* OR neuropath* OR nerve OR neck OR central OR abdominal) N4 pain*) OR AB ((chronic* OR back OR musculoskel* OR muscle OR nocicepti* OR intractabl* OR neuropath* OR nerve OR neck OR central OR abdominal) N4 pain*)

S7 S5 OR S6

S8 MH “psychosocial”

S9 TI (psychosocial OR psycholog* OR mood OR sleep OR “mental disorder” OR “mental illness” OR depress* OR anxiety OR stress OR distress OR posttrauma* OR post-trauma* OR anger OR fatigue) OR AB (psychosocial OR psycholog* OR mood OR sleep OR “mental disorder” OR “mental illness” OR depress* OR anxiety OR stress OR distress OR posttrauma* OR post-trauma* OR anger OR fatigue)

S10 TI (“social support” OR catastrophi* OR cogniti* OR coping OR cope OR accept* OR satisfaction OR self-efficacy OR resilience) OR AB (“social support” OR catastrophi* OR cogniti* OR coping OR cope OR accept* OR satisfaction OR self-efficacy OR resilience)

S11 S8 OR S9 OR S10

S12 S4 AND S7 AND S11

***EMBASE (via Ovid)***

1 exp spinal cord injury/

2 ((spinal cord OR spinal OR spine OR cervical) adj4 (injur* OR lesion* OR trauma* OR transection* OR laceration* OR damage* OR fracture* OR contusion*)).tw

3 (transverse lesion OR transverse cord lesion OR traumatic myelopathy OR paraplegi* OR quadriplegi* OR tetraplegi* OR SCI).tw

4 or/1-3

5 exp pain/

6 ((chronic* OR back OR musculoskel* OR muscle OR nocicepti* OR intractabl* OR neuropath* OR nerve OR neck OR central OR abdominal) adj4 pain*).tw

7 or/5-6

8 exp psychosocial/

9 (psychosocial OR psycholog* OR mood OR sleep OR mental disorder OR mental illness OR depress* OR anxiety OR stress OR distress OR posttrauma* OR post-trauma* OR anger OR fatigue).tw

10 (social support OR catastrophi* OR cogniti* OR coping OR cope OR accept* OR satisfaction OR self-efficacy OR resilience).tw

11 or/8-10

12 4 and 7 and 11
