# Supplementary material for: Psychosocial factors associated with pain in spinal cord injury: a systematic review and meta-analysis
Source: eClinicalMedicine. 2026 May 18;96:103976. doi: 10.1016/j.eclinm.2026.103976 (PMC13316355; doi:10.1016/j.eclinm.2026.103976)
Supplement: Appendix 2 - Study Quality [file mmc2.docx]

**Appendix 2 – Study Quality**

**eTable 1.** Quality assessment tool, adapted from the US National Institutes of Health (NIH).

| **1** | **Study objective** | Was the research question or study objective clearly stated? |
| --- | --- | --- |
| **2** | **Target population** | Was the study population clearly specified and defined? (Clear eligibility criteria) |
| **3** | **Recruitment** | Were all the participants selected or recruited from the same or similar population (including the same time period)? |
| **4** | **Response rate** | Was the participation rate of eligible persons at least 80%? |
| **5** | **Sample size** | Was a sample size justification provided? |
| **6** | **Demographics** | Were all the following characteristics reported? Age, sex, time since injury, and medication use/involvement in any therapies. |
| **7** | **Pain characteristics** | Were all the following pain characteristics reported? Pain duration, pain intensity, and pain location. |
| **8** | **Assessment methods** | Was there a standardised method of data collection across all participants and were data collected directly from participant? |
| **9** | **Assessment tools** | Were the assessment tools previously validated or if a new measure is used, was the reliability and validity reported? |
| **10** | **Statistical analyses** | Were appropriate statistical analyses conducted and measures of association with *p* values and confidence intervals reported? |

NIH. Quality Assessment Tool for Observational Cohort and Cross-Sectional Studies. National Institutes of Health. Accessed Feb 2025, <https://www.nhlbi.nih.gov/health-topics/study-quality-assessment-tools>

**eTable 2.** Quality assessment of each study included in the review using the NIH quality assessment tool, detailed in eTable 1.

| **Study ID** | **1** | **2** | **3** | **4** | **5** | **6** | **7** | **8** | **9** | **10*** | **Overall** |
| --- | --- | --- | --- | --- | --- | --- | --- | --- | --- | --- | --- |
| Alschuler 2013 | Yes | Yes | Yes | Yes | No | No | No | Yes | Yes | Yes | 70% - M |
| Aparicio 2024 | Yes | Yes | Yes | Yes | No | No | No | Yes | Yes | Other | 67% - M |
| Avluk 2014 | Yes | Yes | Yes | No | No | No | No | Yes | Yes | Yes | 60% - M |
| Battalio 2018 | Yes | Yes | Yes | Yes | No | No | No | Yes | Yes | Other | 67% - M |
| Bhattarai 2024 | Yes | Yes | Yes | Yes | No | No | No | Yes | Yes | Yes | 70% - M |
| Bombardier 2023 | Yes | Yes | Yes | Other | Yes | Yes | No | Yes | Yes | Other | 78% - M |
| Bombardier 2024 | Yes | Yes | Yes | Other | No | Yes | Yes | Yes | Yes | Other | 78% - M |
| Braunwalder 2021 | Yes | Yes | Yes | No | No | Yes | No | Yes | Yes | Yes | 60% - M |
| Budh 2005 | Yes | No | Yes | Yes | No | No | No | Yes | Other | Other | 44% - L |
| Burke 2019 | Yes | Yes | Yes | No | Yes | Yes | Yes | Yes | Yes | Other | 89% - H |
| Carlozzi 2022 | Yes | Yes | Yes | Other | Other | No | No | Yes | Yes | Yes | 60% - M |
| Chalageri 2021 | Yes | Yes | Yes | No | No | No | No | Yes | Yes | Other | 56% - M |
| Cherup 2025 | Yes | Yes | Yes | Other | No | No | Yes | Yes | Yes | Other | 67% - M |
| Christofi 2023 | Yes | Yes | Yes | Yes | No | No | No | Yes | Yes | Yes | 70% - M |
| Conant 1998 | Yes | Yes | Yes | No | No | No | Yes | Yes | Yes | Yes | 70% - M |
| Craig 2013 | Yes | Yes | Yes | Other | No | Yes | No | Yes | Yes | Yes | 70% - M |
| Craig 2014 | Yes | Yes | Yes | Other | No | Yes | No | Yes | Yes | Yes | 70% - M |
| Craig 2017 | Yes | Yes | Yes | Yes | Other | Yes | No | Yes | Yes | Yes | 80% - H |
| Craig 2020 | Yes | Yes | Yes | Other | Other | No | No | Yes | Yes | Yes | 60% - M |
| Curtis 2017 | Yes | Yes | Yes | No | Other | No | No | Yes | Yes | Yes | 60% - M |
| Dear 2018 | Yes | Yes | Yes | Yes | No | Yes | Yes | Yes | Yes | Yes | 90% - H |
| DeShazo 2024 | Yes | Yes | Yes | Yes | No | No | No | Yes | No | Other | 56% - M |
| de la Vega 2019 | Yes | Yes | Yes | No | No | No | Yes | Yes | Yes | Yes | 70% - M |
| Finley 2020 | Yes | Yes | Yes | Other | No | No | No | Yes | Yes | Yes | 60% - M |
| Finnerup 2016 | Yes | No | Yes | Yes | No | Yes | Yes | Yes | Yes | Other | 78% - M |
| Forwell 2017 | Yes | Yes | Yes | No | No | No | No | Yes | Yes | Yes | 60% - M |
| Gee 2022 | Yes | Yes | Yes | No | No | No | No | Yes | Yes | Yes | 60% - M |
| Giardino 2003 | Yes | Yes | Yes | Other | No | No | No | Yes | Yes | Yes | 60% - M |
| Goraczko 2021 | Yes | Yes | Yes | No | No | No | No | Yes | Yes | Yes | 60% - M |
| Hartoonian 2014 | Yes | Yes | Yes | Other | No | No | No | No | Yes | Yes | 50% - M |
| Heutink 2013 | Yes | Yes | Yes | No | No | No | Yes | Yes | Yes | Yes | 70% - M |
| Hilton 2017 | Yes | Yes | Yes | No | No | No | No | Yes | Yes | Yes | 60% - M |
| Hughes 2001 | Yes | Yes | Yes | No | No | No | No | Yes | Yes | Yes | 60% - M |
| Jensen 2007 | Yes | No | Yes | No | No | No | No | Yes | Yes | Yes | 50% - M |
| Jindal 2023 | Yes | Yes | Yes | No | No | No | No | Yes | Yes | Yes | 60% - M |
| Jørgensen 2026 | Yes | Yes | Yes | No | No | Yes | No | Yes | Yes | Other | 60% - M |
| Karran 2023 | Yes | Yes | Yes | Other | No | Yes | Yes | Yes | Yes | Yes | 80% - H |
| Kemp 2014 | Yes | No | Yes | Other | No | No | No | Yes | Other | Yes | 40% - L |
| Khazaeipour 2017 | Yes | Yes | Yes | Other | No | Yes | Yes | Yes | Yes | Yes | 80% - H |
| Kilic 2013 | Yes | Yes | Yes | No | Yes | No | No | Yes | Yes | Yes | 70% - M |
| Kovacs 2016 | Yes | Yes | Yes | No | No | No | No | Yes | Yes | No | 50% - M |
| Kratz 2017 | Yes | Yes | Yes | Other | Yes | No | No | Yes | Yes | Yes | 70% - M |
| Krause 2025a | Yes | Yes | Yes | No | No | Yes | No | Yes | Yes | Yes | 70% - M |
| Krause 2025b | Yes | Yes | Yes | No | No | Yes | No | Yes | Yes | Yes | 70% - M |
| Kuiper 2021 | Yes | Yes | Yes | No | No | No | No | Yes | Yes | Yes | 60% - M |
| Li 2022 | Yes | Yes | Yes | Other | No | Yes | No | Yes | Yes | Yes | 70% - M |
| Li 2024 | Yes | Yes | Yes | Yes | No | Yes | No | Yes | Yes | Other | 78% - M |
| Liu 2026 | Yes | Yes | Yes | No | No | No | No | Yes | Yes | Yes | 60% - M |
| Martins Braga 2025 | Yes | Yes | Yes | No | No | Yes | No | Yes | Yes | Other | 67% - M |
| Miro 2014 | Yes | Yes | Yes | No | No | No | Yes | Yes | Other | Yes | 60% - M |
| Morse 2025 | Yes | Yes | Yes | No | No | Yes | Yes | Yes | Yes | Other | 78% - M |
| Muller 2017 | Yes | Yes | Yes | No | No | No | No | Yes | Yes | Yes | 60% - M |
| Murray 2017 | Yes | Yes | Yes | No | No | Yes | Yes | Yes | Yes | Other | 78% - M |
| Nicholson Perry 2009a | Yes | Yes | Yes | No | No | No | No | Yes | Yes | Yes | 60% - M |
| Nicholson Perry 2009b | Yes | Yes | Yes | Other | No | No | No | Yes | Yes | Yes | 60% - M |
| Ratcliff 2024 | Yes | Yes | Yes | Other | No | No | No | Yes | Yes | Yes | 60% - M |
| Rintala 1998 | Yes | Yes | Yes | No | No | No | Yes | Yes | Yes | Yes | 70% - M |
| Rintala 2004 | Other | Yes | Yes | Other | No | Yes | No | Yes | Yes | Other | 56% - M |
| Robinson-Whelen 2014 | Yes | Yes | Yes | No | No | No | No | Yes | Yes | Yes | 60% - M |
| Rodrigues 2013 | Yes | Yes | Yes | Other | No | Yes | No | Yes | Yes | Other | 56% - M |
| Siddall 2017 | Yes | Yes | Other | No | No | No | No | Yes | Yes | Other | 44% - L |
| Summers 1991 | Yes | Yes | Yes | No | No | No | No | Yes | Yes | Yes | 60% - M |
| Trost 2022 | Yes | Yes | Yes | Other | No | Yes | Yes | Yes | Yes | Other | 78% - M |
| Uhlig-Reche 2025 | Yes | Yes | Yes | No | No | No | No | Yes | Yes | Other | 50% - M |
| Ullrich 2007 | Yes | No | Other | No | No | No | No | Yes | Yes | Yes | 40% - L |
| Ullrich 2008 | Yes | No | Yes | No | No | No | No | Yes | Yes | Yes | 50% - M |
| Ullrich 2013 | Yes | No | Yes | No | No | No | No | Yes | Yes | Yes | 50% - M |
| van de Winckel 2023 | Yes | Yes | Yes | Yes | Other | Yes | No | Yes | Yes | Other | 78% - M |
| van Lankveld 2011 | Yes | Yes | Yes | No | No | No | No | Yes | Yes | Yes | 60% - M |
| van Leeuwen 2012 | Yes | Yes | Yes | Other | No | No | No | Yes | Other | Other | 44% - L |
| Vassend 2011 | Yes | Yes | Yes | Yes | No | No | No | Yes | Yes | Other | 67% - M |
| Vives Alvarado 2022 | Yes | Yes | Yes | Yes | No | No | No | Yes | Yes | Other | 67% - M |
| Voerman 2010 | Yes | Yes | Yes | Yes | No | No | No | Yes | Yes | Yes | 70% - M |
| Watson 2022 | Yes | No | Yes | Other | No | No | No | No | Yes | Yes | 40% - L |
| Wen 2013 | Yes | Yes | Yes | Yes | No | Yes | Yes | Yes | Yes | Yes | 90% - H |
| Williams 2024 | Yes | No | Yes | Other | No | Yes | Yes | No | Yes | Yes | 60% - M |
| Wilson 2005 | Yes | Yes | Yes | No | No | No | No | Yes | Yes | Yes | 60% - M |
| Wollaars 2007 | Yes | Yes | Yes | No | No | Yes | Yes | Yes | Yes | Yes | 80% - H |

Each item of the quality assessment was rated as Yes (1), No (0), or Other (not applicable or cannot determine). A total quality score was calculated for each study, with ratings classified into three categories: **L: low** (<50%), **M: medium** (50%-79%), and **H: high** (≥80%) quality.

* For studies where raw data were analysed by NH-S, the statistical analysis criterion was marked as ‘other’; this did not impact the overall quality rating of those studies.
