# Supplementary material for: Psychosocial factors associated with pain in spinal cord injury: a systematic review and meta-analysis
Source: eClinicalMedicine. 2026 May 18;96:103976. doi: 10.1016/j.eclinm.2026.103976 (PMC13316355; doi:10.1016/j.eclinm.2026.103976)
Supplement: Appendix 3 - Study Characteristics [file mmc3.docx]

**Appendix 3 – Study Characteristics**

**eTable 3.** Characteristics of the 78 included studies: design, country, sample size, percentage of female, age, and time since injury.

| **Study ID** | **Design** | **Country** | **Sample size** | **Female, %** | **Age, years (SD)** | **Time since injury, years (SD)** |
| --- | --- | --- | --- | --- | --- | --- |
| Alschuler 2013 ^1^ | Cross-sectional | United States | 481 | 0.33 | 50.0 (14.0) | 17.5 (11.9) |
| Aparicio 2024 ^2^ | Cross-sectional | Switzerland | 549 | 0.25 | 47.7 (10.3) | 18.2 (11.2) |
| Avluk 2014 ^3^ | Cross-sectional | Turkey | 44 | 0.25 | 33.9 (12.7) | *NR* |
| Battalio 2018 ^4^ | Longitudinal | United States | 391 | 0.32 | 53.2 (13.5) | 18.8 (11.5) |
| Bhattarai 2024 ^5^ | Cross-sectional | United States | 272 | 0.35 | 34.7 (9.1) | 6.3 (7.9) |
| Bombardier 2023 ^6^ | RCT | United States | 174 | 0.24 | 47.7 (14.9) | *NR* |
| Bombardier 2024 ^7^ | Non-randomised | United States | 44 | 0.30 | 54.5 (13.7) | 12.4 (10.6) |
| Braunwalder 2021 ^8^ | Cross-sectional | Switzerland | 1064 | 0.31 | 56.9 (14.2) | 19.1 (13.0) |
| Budh 2005 ^9^ | Cross-sectional | Sweden | 141 | 0.47 | 53.8 (28.8) | 15.6 (11.3) |
| Burke 2019 ^10^ | RCT | Ireland | 69 | 0.25 | 51.0 (13.0) | 16.0 (12.1) |
| Carlozzi 2022 ^11^ | Longitudinal | United States | 170 | 0.34 | 49.3 (14.8) | 17.2 (13.6) |
| Chalageri 2021 ^12^ | Non-randomised | India | 91 | 0.15 | 32.8 (11.51) | 4.1 (4.4) |
| Cherup 2025 ^13^ | Quasi-experimental | United States | 36 | 0.25 | 42.3 (14.0) | 10.0 (9.9) |
| Christofi 2023 ^14^ | Cross-sectional | Brazil | 95 | 0.16 | 46.8 (14.7) | 9.1 (8.5) |
| Conant 1998 ^15^ | Cross-sectional | United States | 103 | 0.34 | 42.0 (11.7) | 12.0 (9.4) |
| Craig 2013 ^16^ | Cross-sectional | Australia | 70 | 0.10 | 47.1 (12.0) | 17.4 (14.0) |
| Craig 2014 ^17^ | Cross-sectional | Australia | 107 | 0.13 | 47.1 (14.0) | 14.7 (14.0) |
| Craig 2017 ^18^ | Longitudinal | Australia | 88 | 0.30 | 42.6 (17.8) | 1.0 |
| Craig 2020 ^19^ | Cross-sectional | Australia | 45 | 0.16 | 50.4 (18.0) | 10.4 (13.0) |
| Curtis 2017 ^20^ | RCT | Canada | 22 | *NR* | 51.3 (14.8) | *NR* |
| Dear 2018 ^21^ | Quasi-experimental | Australia | 68 | 0.50 | 48.0 (13.0) | 8.0 (10.0) |
| DeShazo 2024 ^22^ | Longitudinal | Netherlands | 149 | 0.19 | 40.7 (11.8) | 9.5 (10.2) |
| de la Vega 2019 ^23^ | Cross-sectional | United States | 91 | *NR* | 56.2 (11.7) | *NR* |
| Finley 2020 ^24^ | Cross-sectional | United States | 26 | 0.00 | 42.3 (13.6) | 17.3 (12.9) |
| Finnerup 2016 ^25^ | Longitudinal | Denmark | 81 | 0.12 | 51.8 (15.6) | 3.5 (0.6) |
| Forwell 2017 ^26^ | Longitudinal | Canada | 52 | 0.21 | 46.3 (17.8) | 1.0 |
| Gee 2022 ^27^ | RCT | Canada | 35 | 0.26 | 39.0 (11.0) | 11.0 (9.0) |
| Giardino 2003 ^28^ | RCT | United States | 74 | 0.19 | 41.0 (10.0) | 14.0 |
| Goraczko 2021 ^29^ | Cross-sectional | Poland | 9 | *NR* | 38.8 (9.7) | 11.8 (5.2) |
| Hartoonian 2014 ^30^ | Cross-sectional | United States | 4976 | 0.22 | 39.09 (16.39) | *NR* |
| Heutink 2013 ^31^ | RCT | Netherlands | 47 | 0.34 | 58.0 (11.8) | 7.3 |
| Hilton 2017 ^32^ | Longitudinal | Australia | 60 | 0.30 | 35.1 (14.9) | 3.5 (1.1) |
| Hughes 2001 ^33^ | Cross-sectional | United States | 64 | 1.00 | 44.6 (11.7) | 12.5 (10.8) |
| Jensen 2007 ^34^ | Cross-sectional | United States | 147 | 0.25 | 48.8 (13.0) | 16.6 (10.4) |
| Jindal 2023 ^35^ | Cross-sectional | India | 93 | 0.27 | 35 (25-45) * | 4.2 |
| Jørgensen 2026 ^36^ | Quasi-experimental | Norway | 17 | 0.29 | 51.0 (14.0) | 8.0 |
| Karran 2023 ^37^ | Cross-sectional | Australia | 43 | 0.28 | 49.2 (14.4) | 15.8 (15.5) |
| Kemp 2014 ^38^ | Cross-sectional | United States | 134 | 0.35 | 48.8 (12.7) | 24.8 (13.3) |
| Khazaeipour 2017 ^39^ | Cross-sectional | Iran | 70 | 0.30 | 29.4 (8.3) | 3.9 ^o^ |
| Kilic 2013 ^40^ | Cross-sectional | Australia | 60 | 0.32 | 50.8 (17.0) | 5.7 (7.3) |
| Kovacs 2016 ^41^ | Cross-sectional | Spain | 43 | *NR* | *NR* | *NR* |
| Kratz 2017 ^42^ | Cross-sectional | United States | 128 | 0.26 | 47.5 (13.5) | 14.0 (12.2) |
| Krause 2025a ^43^ | Cross-sectional | United States | 918 | 0.30 | 57.5 | 24.4 |
| Krause 2025b ^44^ | Cross-sectional | United States | 918 | 0.30 | 57.5 | 24.4 |
| Kuiper 2021 ^45^ | Cross-sectional | Netherlands | 175 | 0.35 | 53.6 (14.1) | 13.8 (12.8) |
| Li 2022 ^46^ | Cross-sectional | United States | 4670 | 0.26 | 48.8 (15.2) | 11.4 (9.7) |
| Li 2024 ^47^ | RCT | China | 72 | 0.56 | 57.6 (8.7) | 24.0 ^o^ |
| Liu 2026 ^48^ | Cross-sectional | China | 122 | 0.26 | 36.0 | 2.1 |
| Martins Braga 2025 ^49^ | Longitudinal | Spain | 15 | 0.07 | 47.4 (13.7) | 8.9 (10.5) |
| Miro 2014 ^50^ | Cross-sectional | United States | 259 | 0.28 | 46.7 (13.2) | *NR* |
| Morse 2025 ^51^ | RCT | United States | 20 | 0.30 | 47.1 (11.0) | 14.0 (9.7) |
| Muller 2017 ^52^ | Cross-sectional | Switzerland | 1549 | 0.29 | 52.0 (42-63) * | 13.5 |
| Murray 2017 ^53^ | Cross-sectional | United States | 417 | 0.41 | 35.1 (8.4) | 21.0 (9.3) |
| Nicholson Perry 2009a ^54^ | Cross-sectional | Australia | 47 | 0.17 | 39.8 (15.1) | *NR* |
| Nicholson Perry 2009b ^55^ | Cross-sectional | Australia | 45 | 0.16 | 46.5 (16.2) | 6.6 (8.9) |
| Ratcliff 2024 ^56^ | Cross-sectional | United States | 64 | 0.31 | 46.7 (14.1) | 13.6 (16.9) |
| Rintala 1998 ^57^ | Cross-sectional | United States | 58 | 0.00 | 41.0 (12.3) | 13.3 (8.6) |
| Rintala 2004 | Longitudinal | United States | 166 | 0.24 | 44.3 (12.9) | 15.2 (9.7) |
| Robinson-Whelen 2014 ^58^ | Cross-sectional | United States | 51 | 1.00 | 46.6 (12.6) | 12.6 (12.7) |
| Rodrigues 2013 ^59^ | Cross-sectional | Australia | 107 | 0.13 | 47.1 (14.4) | 14.7 (13.9) |
| Siddall 2017 ^60^ | Cross-sectional | Australia | 53 | 0.26 | 55.7 (13.8) | *NR* |
| Summers 1991 ^61^ | Cross-sectional | United States | 54 | 0.17 | 40.5 (11.8) | 12.0 (8.0) |
| Trost 2022 ^62^ | Non-randomised | United States | 28 | 0.18 | 43.0 (12.5) | 11.7 (9.3) |
| Uhlig-Reche 2025 ^63^ | Quasi-experimental | United States | 10 | 0.2 | 45.0 (14.0) | 9.2 (10.5) |
| Ullrich 2007 ^64^ | Cross-sectional | United States | 237 | *NR* | 46.4 (13.3) ^+^ | 10.7 (9.8) ^+^ |
| Ullrich 2008 ^65^ | Cross-sectional | United States | 132 | 0.00 | 56.5 (12.8) | 18.9 (12.6) |
| Ullrich 2013 ^66^ | Longitudinal | United States | 286 | 0.03 | 53.0 | 17.0 |
| van de Winckel 2023 ^67^ | Longitudinal | United States | 18 | 0.33 | 59.6 (11.5) | 15.2 (11.2) |
| van Lankveld 2011 ^68^ | Cross-sectional | Netherlands | 130 | *NR* | 56.2 (15.8) | 8.7 |
| van Leeuwen 2012 ^69^ | Longitudinal | Netherlands | 154 | 0.28 | 41.5 (14.2) | 2.0 |
| Vassend 2011 ^70^ | Longitudinal | Norway | 75 | 0.33 | 45.0 (14.5) | 4.3 |
| Vives Alvarado 2022 ^71^ | Cross-sectional | United States | 51 | 0.06 | 41.7 (14.3) | 4.3 (4.7) |
| Voerman 2010 ^72^ | Cross-sectional | Netherlands | 19 | 0.21 | 41.2 (25.7-62.6) * | 9.6 |
| Watson 2022 ^73^ | Cross-sectional | United States | 221 | 0.03 | 58.6 (12.7) | 16.4 (13.7) |
| Wen 2013 ^74^ | Longitudinal | China | 26 | 0.54 | 50.8 (14.7) | *NR* |
| Williams 2024 ^75^ | Cross-sectional | South Africa | 70 | 0.13 | 35.5 (9.3) | *NR* |
| Wilson 2005 ^76^ | Longitudinal | United States | 1334 | 0.21 | 44.5 (11.9) | *NR* |
| Wollaars 2007 ^77^ | Cross-sectional | Netherlands | 279 | 0.36 | 51.1 (14.2) | 11.8 (10.7) |

* Age and time since injury are presented as mean (SD) unless otherwise indicated by *, which represents median (IQR).

^+^ Estimated mean (SD) for age and time since injury, calculated assuming an equal distribution of females and males. Ullrich 2007 reported age and time since injury separately by sex but did not provide the proportion of female/male participants.

^o^ Some studies did not report SD for mean age and/or time since injury.

**RCT:** Randomised Controlled Trial**, NR:** not reported.

**References**

1. Alschuler KN, Jensen MP, Sullivan-Singh SJ, Borson S, Smith AE, Molton IR. The association of age, pain, and fatigue with physical functioning and depressive symptoms in persons with spinal cord injury. *J Spinal Cord Med*. 2013;36(5):483-491.

2. Aparicio MG, Mwake I, Ronca-Nützi M, Staubli S, Schwegler U. Perceived job quality among persons with spinal cord injury: The contribution of sociodemographic characteristics, health-related factors, and person-job match. *J Spinal Cord Med*. 2024;47(5):733-743.

3. Avluk OC, Gurcay E, Gurcay AG, Karaahmet OZ, Tamkan U, Cakci A. Effects of chronic pain on function, depression, and sleep among patients with traumatic spinal cord injury. *Ann Saudi Medicine*. 2014;34(3):211-216.

4. Battalio SL, Glette M, Alschuler KN, Jensen MP. Anxiety, depression, and function in individuals with chronic physical conditions: A longitudinal analysis. *Rehabil psychol*. 2018;63(4):532.

5. Bhattarai M, McDaniels B, Jin Y, Smedema SM. Pain and quality of life in persons with spinal cord injury: Mediating effects of mindfulness, self‐efficacy, social support, and functional independence. *J Clin Psychol*. 2024;80(2):406-420.

6. Bombardier CH, Fann JR, Ehde DM, et al. Collaborative care versus usual care to improve quality of life, pain, depression, and physical activity in outpatients with spinal cord injury: the SCI-CARE randomized controlled clinical trial. *J Neurotrauma*. 2023;40(23-24):2667-2679.

7. Bombardier CH, Chan JF, Stensland E, Barber J, Jensen MP. The efficacy, safety, and satisfaction of telehealth-delivered hypnotic cognitive therapy for chronic pain in spinal cord injury: A pilot study with historical controls. *J Spinal Cord Med*. 2024:1-12.

8. Braunwalder C, Müller R, Kunz S, Tough H, Landmann G, Fekete C. Psychosocial resources and chronic pain in individuals with spinal cord injury: evidence from the second Swiss national community survey. *Spinal Cord*. 2021;59(4):410-418.

9. Budh C, Hultling C, Lundeberg T. Quality of sleep in individuals with spinal cord injury: a comparison between patients with and without pain. *Spinal cord*. 2005;43(2):85-95.

10. Burke D, Lennon O, Blake C, et al. An internet‐delivered cognitive behavioural therapy pain management programme for spinal cord injury pain: A randomized controlled trial. *Eur J Pain*. 2019;23(7):1264-1282.

11. Carlozzi NE, Freedman J, Troost JP, et al. Daily variation in sleep quality is associated with health-related quality of life in people with spinal cord injury. *Arch Phys Med Rehabil*. 2022;103(2):263-273. e4.

12. Chalageri E, Vishwakarma G, Ranjan RL, Govindaraj R, Chhabra HS. Effect of Rāja yoga meditation on psychological and functional outcomes in spinal cord injury patients. *Int J Yoga*. 2021;14(1):36-42.

13. Cherup NP, Anderson KD, Wong ML, et al. Impact of a pain education program for people with spinal cord injury who experience neuropathic pain. *Frontiers in Pain Research*. 2025;6:1569446.

14. Christofi AA, Tate DG, Witter C, Alonso AC, Greve JMDA. Predictors of quality of life of individuals living in Brazil with spinal cord injury/disease. *Spinal Cord*. 2023;61(4):253-259.

15. Conant LL. Psychological variables associated with pain perceptions among individuals with chronic spinal cord injury pain. *J Clin Psychol Med Settings*. 1998;5:71-90.

16. Craig A, Tran Y, Siddall P, et al. Developing a model of associations between chronic pain, depressive mood, chronic fatigue, and self-efficacy in people with spinal cord injury. *J Pain*. 2013;14(9):911-920.

17. Craig A, Rodrigues D, Tran Y, Guest R, Bartrop R, Middleton J. Developing an algorithm capable of discriminating depressed mood in people with spinal cord injury. *Spinal Cord*. 2014;52(5):413-416.

18. Craig A, Guest R, Tran Y, Perry KN, Middleton J. Pain catastrophizing and negative mood states after spinal cord injury: transitioning from inpatient rehabilitation into the community. *J Pain*. 2017;18(7):800-810.

19. Craig A, Tran Y, Guest R, Middleton J. Excessive daytime sleepiness in adults with spinal cord injury and associations with pain catastrophizing and pain intensity. *Spinal Cord*. 2020;58(7):831-839.

20. Curtis K, Hitzig SL, Bechsgaard G, et al. Evaluation of a specialized yoga program for persons with a spinal cord injury: a pilot randomized controlled trial. *J Pain Res*. 2017:999-1017.

21. Dear B, Nicholson Perry K, Siddall P, et al. The Pain Course: Exploring the feasibility of an internet-delivered pain management programme for adults with spinal cord injury. *Spinal Cord*. 2018;56(10):931-939.

22. DeShazo JM, Kouwijzer I, de Groot S, et al. Effect of Training for an Athletic Challenge on Illness Cognition in Individuals with Chronic Disability: A Prospective Cohort Study. *Int J Environ Res Public Health*. 2023;21(1):58.

23. de la Vega R, Miró J, Esteve R, Ramírez-Maestre C, López-Martínez AE, Jensen MP. Sleep disturbance in individuals with physical disabilities and chronic pain: The role of physical, emotional and cognitive factors. *Disabil Health J*. 2019;12(4):588-593.

24. Finley MA, Euiler E. Association of musculoskeletal pain, fear-avoidance factors, and quality of life in active manual wheelchair users with SCI: A pilot study. *J Spinal Cord Med*. 2020;43(4):497-504.

25. Finnerup N, Jensen MP, Norrbrink C, et al. A prospective study of pain and psychological functioning following traumatic spinal cord injury. *Spinal Cord*. 2016;54(10):816-821.

26. Forwell SJ, Silverberg ND, Anton HA, et al. Fatigue, pain, and depression: an invisible triad among persons with spinal cord injury. *Phys Ther Rev*. 2017;22(1-2):7-11.

27. Gee CM, Sinden AR, Krassioukov AV, Martin Ginis KA. The effects of active upper-limb versus passive lower-limb exercise on quality of life among individuals with motor-complete spinal cord injury. *Spinal cord*. 2022;60(9):805-811.

28. Giardino ND, Jensen MP, Turner JA, Ehde DM, Cardenas DD. Social environment moderates the association between catastrophizing and pain among persons with a spinal cord injury. *Pain*. 2003;106(1-2):19-25.

29. Goraczko A, Zurek A, Lachowicz M, Kujawa K, Zurek G. Is self-efficacy related to the quality of life in elite athletes after spinal cord injury? *Int J Environ Res Public Health*. 2021;18(20):10866.

30. Hartoonian N, Hoffman JM, Kalpakjian CZ, Taylor HB, Krause JK, Bombardier CH. Evaluating a spinal cord injury–specific model of depression and quality of life. *Arch Phys Med Rehabil*. 2014;95(3):455-465.

31. Heutink M, Post M, Overdulve C, et al. Which pain coping strategies and cognitions are associated with outcomes of a cognitive behavioral intervention for neuropathic pain after spinal cord injury? *Top Spinal Cord Inj Rehabil*. 2013;19(4):330-340.

32. Hilton G, Unsworth CA, Murphy G, Browne M, Olver J. Longitudinal employment outcomes of an early intervention vocational rehabilitation service for people admitted to rehabilitation with a traumatic spinal cord injury. *Spinal Cord*. 2017;55(8):743-752.

33. Hughes R, Swedlund N, Petersen N, Nosek M. Depression and women with spinal cord injury. *Top Spinal Cord Inj Rehabil*. 2001;7(1):16-24.

34. Jensen MP, Kuehn CM, Amtmann D, Cardenas DD. Symptom burden in persons with spinal cord injury. *Arch Phys Med Rehabil*. 2007;88(5):638-645.

35. Jindal R, Bansal P, Gupta S, Garg SK. Quality of life after traumatic thoracolumbar spinal cord injury: a North Indian perspective. *Spinal Cord*. 2023;61(7):374-382.

36. Jørgensen V, Flaaten AB, Ingvarsson PE, Lannem AM. Patient-reported effects of transcutaneous spinal cord stimulation on spasticity in patients with spinal cord injury. *The Journal of Spinal Cord Medicine*. 2026;49(1):154-161.

37. Karran EL, Fryer CE, Middleton JW, Moseley GL. Pain and pain management experiences following spinal cord injury–a mixed methods study of Australian community-dwelling adults. *Disabil Rehabil*. 2023;45(3):455-468.

38. Kemp B, Tsukerman D, Kahan J, Adkins R. Predicting psychosocial outcomes using a brief measure of quality of life in a sample of people with spinal cord injury. *Top Spinal Cord Inj Rehabil*. 2014;20(3):191-196.

39. Khazaeipour Z, Ahmadipour E, Rahimi-Movaghar V, Ahmadipour F, Vaccaro A, Babakhani B. Association of pain, social support and socioeconomic indicators in patients with spinal cord injury in Iran. *Spinal cord*. 2017;55(2):180-186.

40. Kilic S, Dorstyn D, Guiver N. Examining factors that contribute to the process of resilience following spinal cord injury. *Spinal cord*. 2013;51(7):553-557.

41. Kovacs F, Barriga A, Royuela A, Seco J, Zamora J. Spanish adaptation of the quality of life index-spinal cord injury version. *Spinal Cord*. 2016;54(10):895-900.

42. Kratz AL, Ehde DM, Bombardier CH, Kalpakjian CZ, Hanks RA. Pain acceptance decouples the momentary associations between pain, pain interference, and physical activity in the daily lives of people with chronic pain and spinal cord injury. *J Pain*. 2017;18(3):319-331.

43. Krause JS, Dipiro ND, Dismuke-Greer CE, Laursen-Roesler J. Relationships of self-reported opioid and benzodiazepine use with health-related quality of life among adults with spinal cord injury. *Disability and Health Journal*. 2025;18(1):101668.

44. Krause JS, Dismuke-Greer CE, Grant B. The relationship of resilience with prescription opioid use and misuse among people with spinal cord injury. *Rehabilitation Psychology*. 2025;

45. Kuiper H, van Leeuwen CC, Kopsky DJ, Stolwijk-Swüste JM, Post MW. Post-traumatic stress disorder symptoms and pain intensity in persons with spinal cord injury. *Spinal cord*. 2021;59(3):328-335.

46. Li C, DiPiro ND, Clark JMR, Krause JS. Mediating Effects of Pain Interference on the Relationships Between Pain Intensity and Probable Major Depression Among Participants With Spinal Cord Injury. *Arch Phys Med Rehabil*. Apr 2022;103(4):747-754. doi:10.1016/j.apmr.2021.04.011

47. Li Y, Li M, Bressington D, et al. Effect of a Mindfulness and Motivational Interviewing-Oriented Physical-Psychological Integrative Intervention for Community-Dwelling Spinal Cord Injury Survivors: A Mixed-Methods Randomized Controlled Trial. *Arch Phys Med Rehabil*. Sep 2024;105(9):1632-1641. doi:10.1016/j.apmr.2024.05.017

48. Liu Y, Luo C, Nie L, et al. Relationships between physical activity and depression in community-dwelling individuals with spinal cord injury: a study based on structural equation modeling and fuzzy-set qualitative comparative analysis. *Disability and Rehabilitation*. 2026:1-10.

49. Martins Braga F, Albu S, Mariño Fernández C, Martínez Santana V, Benito-Penalva J, Vidal Samsó J. The effects of nabiximols (Sativex®) on spasticity and non-motor symptoms in chronic spinal cord injury (SCI): a longitudinal prospective study. *Spinal Cord Series and Cases*. 2025;11(1):19.

50. Miró J, Gertz KJ, Carter GT, Jensen MP. Pain location and functioning in persons with spinal cord injury. *PM R*. Aug 2014;6(8):690-7. doi:10.1016/j.pmrj.2014.01.010

51. Morse LR, Battaglino RA, Nguyen N, et al. Brivaracetam for spinal cord injury–related neuropathic pain: results of a pilot double-blinded, randomized, placebo-controlled clinical trial. *Pain reports*. 2025;10(4):e1301.

52. Müller R, Landmann G, Béchir M, et al. Chronic pain, depression and quality of life in individuals with spinal cord injury: Mediating role of participation. *J Rehabil Med*. Jun 28 2017;49(6):489-496. doi:10.2340/16501977-2241

53. Murray CB, Zebracki K, Chlan KM, Moss AC, Vogel LC. Medical and psychological factors related to pain in adults with pediatric-onset spinal cord injury: a biopsychosocial model. *Spinal Cord*. Apr 2017;55(4):405-410. doi:10.1038/sc.2016.137

54. Nicholson Perry K, Nicholas MK, Middleton J, Siddall P. Psychological characteristics of people with spinal cord injury-related persisting pain referred to a tertiary pain management center. *J Rehabil Res Dev*. 2009;46(1):57-67.

55. Nicholson Perry K, Nicholas MK, Middleton J. Spinal cord injury-related pain in rehabilitation: a cross-sectional study of relationships with cognitions, mood and physical function. *Eur J Pain*. May 2009;13(5):511-7. doi:10.1016/j.ejpain.2008.06.003

56. Ratcliff CG, Lohiya S, Robinson-Whelen S, Taylor H, Ahn A, Korupolu R. Mindfulness buffers the association of pain with depression and anxiety among people with spinal cord injury: A cross-sectional study. *Rehabil Psychol*. Nov 11 2024;doi:10.1037/rep0000593

57. Rintala DH, Loubser PG, Castro J, Hart KA, Fuhrer MJ. Chronic pain in a community-based sample of men with spinal cord injury: prevalence, severity, and relationship with impairment, disability, handicap, and subjective well-being. *Arch Phys Med Rehabil*. Jun 1998;79(6):604-14. doi:10.1016/s0003-9993(98)90032-6

58. Robinson-Whelen S, Taylor HB, Hughes RB, Wenzel L, Nosek MA. Depression and depression treatment in women with spinal cord injury. *Top Spinal Cord Inj Rehabil*. Winter 2014;20(1):23-31. doi:10.1310/sci2001-23

59. Rodrigues D, Tran Y, Wijesuriya N, Guest R, Middleton J, Craig A. Pain intensity and its association with negative mood States in patients with spinal cord injury. *Pain Ther*. Dec 2013;2(2):113-9. doi:10.1007/s40122-013-0017-8

60. Siddall PJ, McIndoe L, Austin P, Wrigley PJ. The impact of pain on spiritual well-being in people with a spinal cord injury. *Spinal Cord*. Jan 2017;55(1):105-111. doi:10.1038/sc.2016.75

61. Summers JD, Rapoff MA, Varghese G, Porter K, Palmer RE. Psychosocial factors in chronic spinal cord injury pain. *Pain*. 1991;47(2):183-189.

62. Trost Z, Anam M, Seward J, et al. Immersive interactive virtual walking reduces neuropathic pain in spinal cord injury: findings from a preliminary investigation of feasibility and clinical efficacy. *Pain*. Feb 1 2022;163(2):350-361. doi:10.1097/j.pain.0000000000002348

63. Uhlig-Reche H, Hoekstra S, Wu Y, et al. The effect of eight weeks of passive heat therapy on mental health, sleep, and chronic pain in persons with spinal cord injury: a pilot study. *Journal of Clinical Medicine*. 2025;14(10):3566.

64. Ullrich P, Jensen M, Loeser J, Cardenas D. Catastrophizing mediates associations between pain severity, psychological distress, and functional disability among persons with spinal cord injury. *Rehabil Psychol*. 2007;52(4):390.

65. Ullrich PM, Jensen MP, Loeser JD, Cardenas DD, Weaver FM. Pain among veterans with spinal cord injury. *J Rehabil Res Dev*. 2008;45(6):793-800. doi:10.1682/jrrd.2008.01.0005

66. Ullrich PM, Lincoln RK, Tackett MJ, Miskevics S, Smith BM, Weaver FM. Pain, depression, and health care utilization over time after spinal cord injury. *Rehabil Psychol*. May 2013;58(2):158-65. doi:10.1037/a0032047

67. Van de Winckel A, Carpentier ST, Deng W, et al. Feasibility of using remotely delivered Spring Forest Qigong to reduce neuropathic pain in adults with spinal cord injury: a pilot study. *Front Physiol*. 2023;14:1222616. doi:10.3389/fphys.2023.1222616

68. van Lankveld W, van Diemen T, van Nes I. Coping with spinal cord injury: tenacious goal pursuit and flexible goal adjustment. *J Rehabil Med*. Oct 2011;43(10):923-9. doi:10.2340/16501977-0870

69. van Leeuwen CM, Post MW, van Asbeck FW, et al. Life satisfaction in people with spinal cord injury during the first five years after discharge from inpatient rehabilitation. *Disabil Rehabil*. 2012;34(1):76-83. doi:10.3109/09638288.2011.587089

70. Vassend O, Quale AJ, Røise O, Schanke AK. Predicting the long-term impact of acquired severe injuries on functional health status: the role of optimism, emotional distress and pain. *Spinal Cord*. Dec 2011;49(12):1193-7. doi:10.1038/sc.2011.70

71. Vives Alvarado JR, Miranda-Cantellops N, Jackson SN, Felix ER. Access limitations and level of psychological distress during the COVID-19 pandemic in a geographically-limited sample of individuals with spinal cord injury. *J Spinal Cord Med*. Sep 2022;45(5):700-709. doi:10.1080/10790268.2021.2013592

72. Voerman GE, Erren-Wolters CV, Fleuren JF, Hermens HJ, Geurts AC. Perceived spasticity in chronic spinal cord injured patients: associations with psychological factors. *Disabil Rehabil*. 2010;32(9):775-80. doi:10.3109/09638280903304490

73. Watson JDK, McDonald SD, Henry RS, Pugh M, Kuzu D, Perrin PB. Pain, mental health, life satisfaction, and understanding from others in veterans with spinal cord injury. *Rehabil Psychol*. Aug 2022;67(3):337-343. doi:10.1037/rep0000430

74. Wen H, Reinhardt JD, Gosney JE, Baumberger M, Zhang X, Li J. Spinal cord injury-related chronic pain in victims of the 2008 Sichuan earthquake: a prospective cohort study. *Spinal Cord*. Nov 2013;51(11):857-62. doi:10.1038/sc.2013.59

75. Williams TL, Joseph C, Nilsson-Wikmar L, Phillips J. The interrelationship between pain, life satisfaction and mental health in adults with traumatic spinal cord injury, in the context of a developing country. *Spinal Cord Ser Cases*. Mar 7 2024;10(1):9. doi:10.1038/s41394-024-00622-9

76. Wilson MW, Richards JS, Klapow JC, DeVivo MJ, Greene P. Cluster Analysis and Chronic Pain: An Empirical Classification of Pain Subgroups in a Spinal Cord Injury Sample. *Rehabil Psychol*. Nov 2005;50(4):381-388. doi:10.1037/0090-5550.50.4.381

77. Wollaars MM, Post MW, van Asbeck FW, Brand N. Spinal cord injury pain: the influence of psychologic factors and impact on quality of life. *Clin J Pain*. Jun 2007;23(5):383-91. doi:10.1097/AJP.0b013e31804463e5
