# Supplementary material for: Psychosocial factors associated with pain in spinal cord injury: a systematic review and meta-analysis
Source: eClinicalMedicine. 2026 May 18;96:103976. doi: 10.1016/j.eclinm.2026.103976 (PMC13316355; doi:10.1016/j.eclinm.2026.103976)
Supplement: Appendix 4 - Statistical Analysis [file mmc4.docx]

**Appendix 4 – Statistical Synthesis**

***Moderators’ Effects***

**eTable 4.** **Overview of moderators’ effects** on the associations between pain intensity and psychosocial factors.

|  | **% Female** | **Age** | **Time since injury** | **% Tetraplegia** | **% Complete injury** | **Scale ranges** |
| --- | --- | --- | --- | --- | --- | --- |
| **Mental Health Factors** | | | | | | |
| **Depression (k = 49)** | NS | NS | NS | NS | NS | NS |
| **Anxiety (k = 23)** | **Weaker** | NS | NS | NS | NS | NS |
| **Psychological Health (k = 18)** | NS | NS | NS | NS | NS | NS |
| **Adaptive Psychological Factors** | | | | | | |
| **Self-efficacy (k = 16)** | NS | **Weaker** | NS | NS | NS | NS |
| **Acceptance (k = 9)** | NS | NS | **Stronger** | NS | NS | NS |
| **Resilience (k = 5)** | NA | NA | NA | NA | NA | NA |
| **Cognitive and Emotional Factors** | | | | | | |
| **Catastrophising (k = 18)** | NS | NS | **Weaker** | NS | NS | NS |
| **Stress (k = 6)** | NA | NA | NA | NA | NA | NA |
| **Anger (k = 5)** | **Weaker** | NS | NS | **Weaker** | NS | **Weaker** |
| **Social and Interpersonal Factors** | | | | | | |
| **Social Functioning (k = 10)** | NA | NA | NA | NA | NA | NA |
| **Social Integration (k = 7)** | NS | NS | NS | NS | NS | NS |
| **Social Support (k = 6)** | **Stronger** | NS | NS | **Weaker** | NS | NS |
| **Fatigue and Sleep** | | | | | | |
| **Fatigue (k = 10)** | NS | NS | NS | NS | NS | **Stronger (pain)**, NS (fatigue) |
| **Sleep disturbance (k = 9)** | NS | NS | NS | NS | NS | NS |
| **Quality of Life and Life Satisfaction** | | | | | | |
| **Quality of life and life satisfaction (k = 16)** | NS | NS | NS | **Weaker** | NS | NS |

**NS:** not significant; **NA:** not applicable

***Forest Plots***


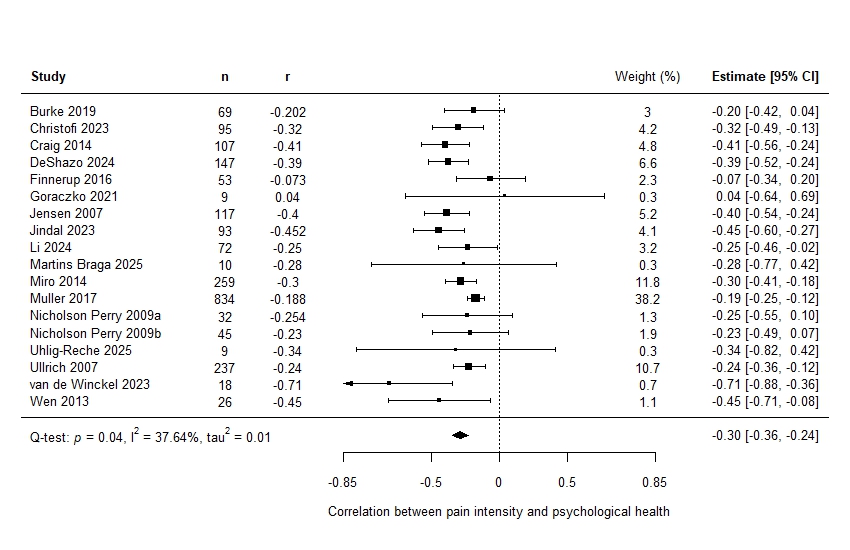


**eFigure 1. Forest plot of pain–psychological health association across 18 studies.**


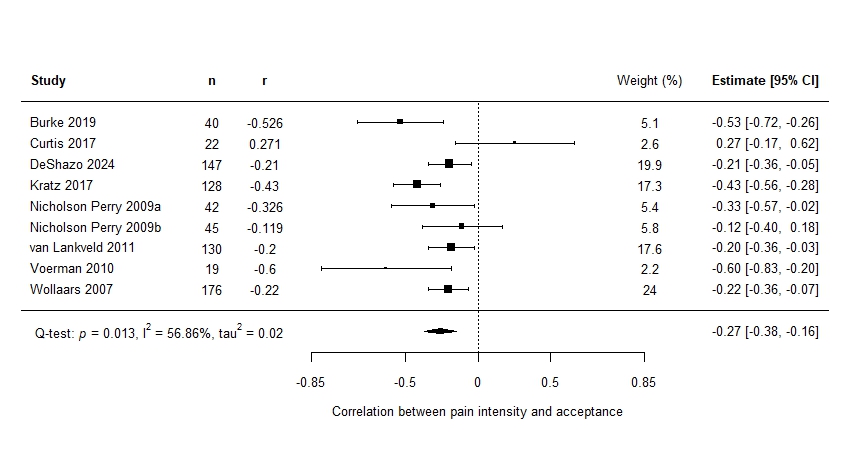


**eFigure 2. Forest plot of pain–acceptance association across nine studies.**


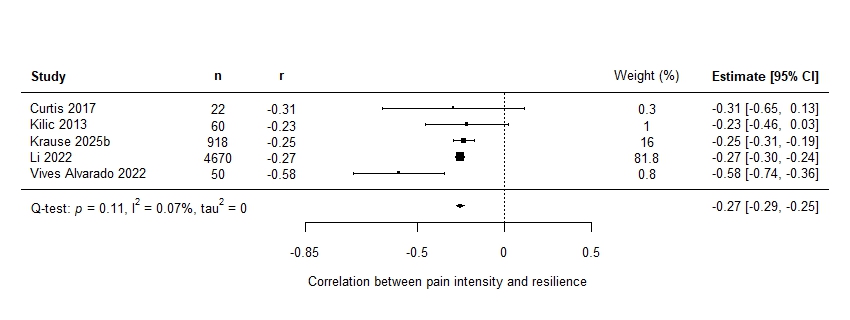


**eFigure 3. Forest plot of pain–resilience association across five studies.**


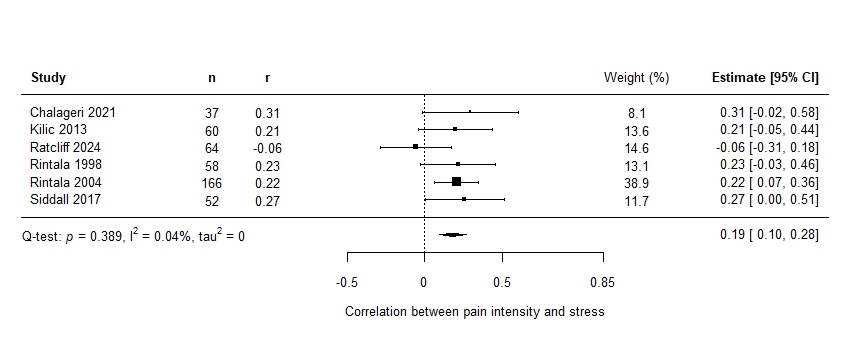


**eFigure 4. Forest plot of pain–stress association across six studies.**


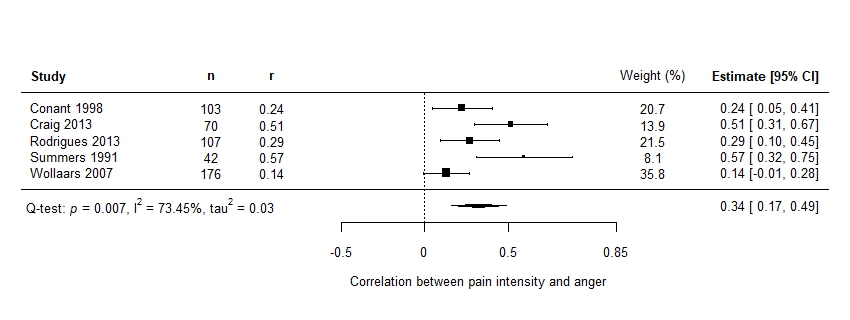


**eFigure 5. Forest plot of pain–anger association across five studies.**


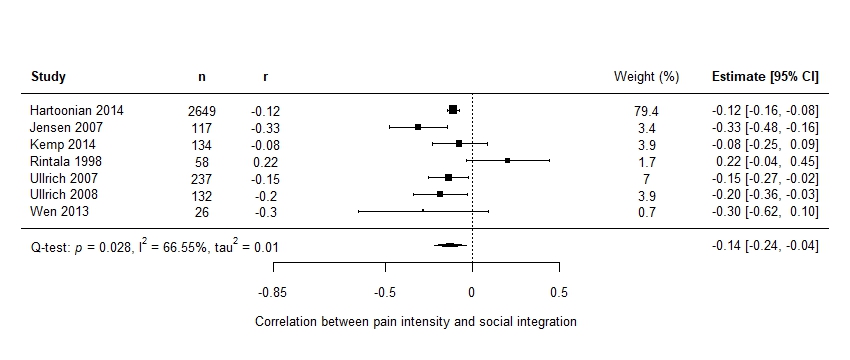


**eFigure 6. Forest plot of pain–social integration association across seven studies.**


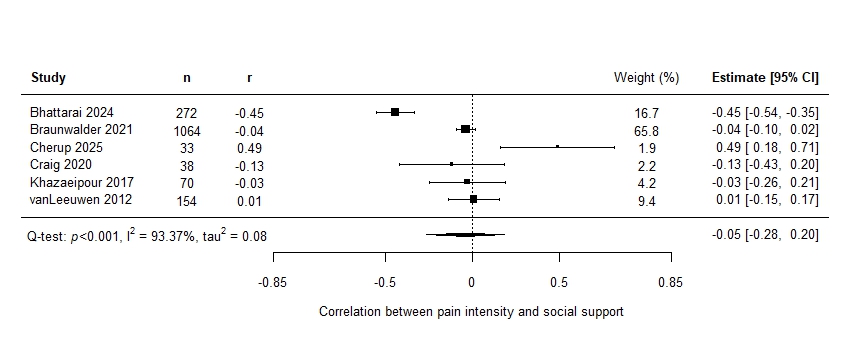


**eFigure 7. Forest plot of pain–social support association across six studies.**


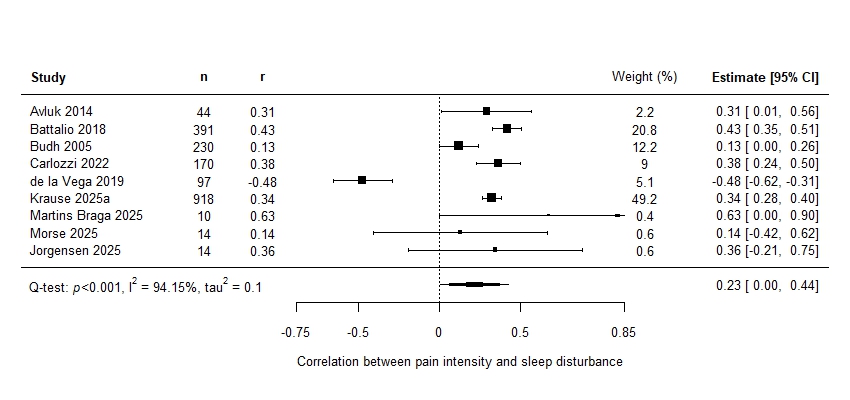


**eFigure 8. Forest plot of pain–sleep association across nine studies.**
