# Supplementary material for: Psychosocial factors associated with pain in spinal cord injury: a systematic review and meta-analysis
Source: eClinicalMedicine. 2026 May 18;96:103976. doi: 10.1016/j.eclinm.2026.103976 (PMC13316355; doi:10.1016/j.eclinm.2026.103976)
Supplement: Appendix 5 - Sensitivity Analysis [file mmc5.docx]

**Appendix 5 – Sensitivity Analyses**

**eTable 5.** **Sensitivity analyses excluding studies not specifying chronic pain.** Original and sensitivity estimates of associations between pain intensity and psychosocial constructs are presented with corresponding changes in heterogeneity.

|  | **Original estimate**  **[95% CI]** | **Sensitivity estimate**  **[95% CI]** | **Compared to original?** | **Sensitivity heterogeneity**  **(Q-test, I²** **and τ²)** | **Compared to original heterogeneity?** |
| --- | --- | --- | --- | --- | --- |
| **Mental Health Factors** |  |  |  |  |  |
| **Depression (k = 14)** | 0.34 [0.30, 0.38] | 0.33 [0.24, 0.41] | *Unchanged/stable* | *p* = .001; I² = 62.89%; τ² = 0.02 | Remained substantial |
| **Anxiety (k = 5)** | 0.34 [0.28, 0.40] | 0.31 [0.18, 0.42] | *Unchanged/stable* | *p* = .660; I² = 0.00%; τ² = 0.00 | Reduced to non-significant |
| **Psychological Health (k = 5)** | -0.30 [-0.36, -0.24] | -0.23 [-0.30, -0.15] | *Weaker* | *p* = .572; I² = 20.29%; τ² = 0.00 | Reduced to non-significant |
| **Adaptive Psychological Factors** |  |  |  |  |  |
| **Self-efficacy (k = 5)** | -0.29 [-0.38, -0.20] | -0.48 [-0.55, -0.41] | *Stronger* | *p* = .621; I² = 0.00%; τ² = 0.00 | Reduced to non-significant |
| **Cognitive and Emotional Factors** |  |  |  |  |  |
| **Catastrophising (k = 9)** | 0.37 [0.29, 0.44] | 0.38 [0.30, 0.46] | *Unchanged/stable* | *p* = .302; I² = 11.36%; τ² = 0.00 | Reduced to non-significant |

**eTable 6. Sensitivity analyses excluding low-quality studies.** Original and sensitivity estimates of associations between pain intensity and psychosocial constructs are presented with corresponding changes in heterogeneity.

|  | **Original estimate**  **[95% CI]** | **Sensitivity estimate**  **[95% CI]** | **Compared to original?** | **Sensitivity heterogeneity**  **(Q-test, I²** **and τ²)** | **Compared to original heterogeneity?** |
| --- | --- | --- | --- | --- | --- |
| **Mental Health Factors** |  |  |  |  |  |
| **Depression (k = 47)** | 0.34 [0.30, 0.38] | 0.34 [0.30, 0.38] | *Unchanged/stable* | *p <* .0001; I² = 64.82%; τ² = 0.01 | Remained substantial |
| **Anxiety (k = 22)** | 0.34 [0.28, 0.40] | 0.34 [0.28, 0.40] | *Unchanged/stable* | *p* = .005; I² = 53.80%; τ² = 0.01 | Remained moderate |
| **Psychological Health (k = 17)** | -0.30 [-0.36, -0.24] | -0.31 [-0.37, -0.24] | *Unchanged/stable* | *p* = .030; I² = 39.02%; τ² = 0.01 | Remained moderate |
| **Adaptive Psychological Factors** |  |  |  |  |  |
| **Self-efficacy (k = 15)** | -0.29 [-0.38, -0.20] | -0.31 [-0.39, -0.22] | *Unchanged/stable* | *p* < .0001; I² = 64.76%; τ² = 0.02 | Remained substantial |
| **Cognitive and Emotional Factors** |  |  |  |  |  |
| **Catastrophising (k = 17)** | 0.37 [0.29, 0.44] | 0.37 [0.29, 0.46] | *Unchanged/stable* | *p* = .002; I² = 56.64%; τ² = 0.02 | Remained substantial |
| **Stress (k = 5)** | 0.19 [0.10, 0.28] | 0.18 [0.07, 0.29] | *Unchanged/stable* | *p* = .318; I² = 9.76%; τ² = 0.00 | Remained non-significant |
| **Social and Interpersonal Factors** |  |  |  |  |  |
| **Social Integration (k = 5)** | -0.14 [-0.24, -0.04] | -0.15 [-0.31, 0.03] | *Became non-significant* | *p* = .008; I² = 81.34%; τ² = 0.03 | Increased to considerable |
| **Fatigue and Sleep** |  |  |  |  |  |
| **Fatigue (k = 9)** | 0.43 [0.31, 0.53] | 0.45 [0.33, 0.55] | *Unchanged/stable* | *p <* .001; I² = 82.48%; τ² = 0.03 | Remained considerable |
| **Quality of Life and Life Satisfaction** |  |  |  |  |  |
| **Quality of life/life satisfaction (k = 12)** | -0.29 [-0.36, -0.22] | -0.30 [-0.42, -0.17] | *Unchanged/stable* | *p* = .010; I² = 71.98%; τ² = 0.03 | Remained moderate |

**eTable 7. Sensitivity analyses excluding intervention-based studies.** Original and sensitivity estimates of associations between pain intensity and psychosocial constructs are presented with corresponding changes in heterogeneity.

|  | **Original estimate**  **[95% CI]** | **Sensitivity estimate**  **[95% CI]** | **Compared to original?** | **Sensitivity heterogeneity**  **(Q-test, I²** **and τ²)** | **Compared to original heterogeneity?** |
| --- | --- | --- | --- | --- | --- |
| **Mental Health Factors** |  |  |  |  |  |
| **Depression (k = 39)** | 0.34 [0.30, 0.38] | 0.34 [0.30, 0.37] | *Unchanged/stable* | *p <* .001; I² = 53.14%; τ² = 0.01 | Remained substantial |
| **Anxiety (k = 22)** | 0.34 [0.28, 0.40] | 0.35 [0.29, 0.41] | *Unchanged/stable* | *p* = .003; I² = 57.93%; τ² = 0.01 | Remained moderate |
| **Psychological Health (k = 15)** | -0.30 [-0.36, -0.24] | -0.31 [-0.38, -0.24] | *Unchanged/stable* | *p* = .014; I² = 45.38%; τ² = 0.01 | Remained moderate |
| **Adaptive Psychological Factors** |  |  |  |  |  |
| **Self-efficacy (k = 13)** | -0.29 [-0.38, -0.20] | -0.28 [-0.37, -0.18] | *Unchanged/stable* | *p* < .0001; I² = 72.02%; τ² = 0.02 | Remained substantial |
| **Acceptance (k = 7)** | -0.27 [-0.38, -0.16] | -0.27 [-0.36, -0.18] | *Unchanged/stable* | *p* = .128; I² = 34.15%; τ² = 0.01 | Reduced to non-significant |
| **Cognitive and Emotional Factors** |  |  |  |  |  |
| **Catastrophising (k = 17)** | 0.37 [0.29, 0.44] | 0.39 [0.29, 0.48] | *Unchanged/stable* | *p* = .003; I² = 65.68%; τ² = 0.02 | Remained substantial |
| **Stress (k = 5)** | 0.19 [0.10, 0.28] | 0.18 [0.08, 0.28] | *Unchanged/stable* | *p* = .343; I² = 3.67%; τ² = 0.00 | Remained non-significant |
| **Social and Interpersonal Factors** |  |  |  |  |  |
| **Social Functioning (k = 8)** | -0.28 [-0.34, -0.22] | -0.30 [-0.37, 0.23] | *Unchanged/stable* | *p* = .714; I² = 0.00%; τ² = 0.00 | Remained non-significant |
| **Social Support (k = 5)** | -0.05 [-0.28, 0.20] | -0.14 [-0.32, 0.06] | *Stronger, still non-significant* | *p* < .0001; I² = 89.22%; τ² = 0.04 | Remained considerable |
| **Fatigue and Sleep** |  |  |  |  |  |
| **Sleep (k = 8)** | 0.23 [0.00, 0.44] | 0.24 [-0.01, 0.46] | *Became non-significant* | *p <* .0001; I² = 95.27%; τ² = 0.11 | Remained considerable |
| **Quality of Life and Life Satisfaction** |  |  |  |  |  |
| **Quality of life/life satisfaction (k = 13)** | -0.29 [-0.36, -0.22] | -0.30 [-0.36, -0.24] | *Unchanged/stable* | *p* = .108; I² = 28.67%; τ² = 0.00 | Reduced to non-significant |
