# Supplementary material for: Psychosocial factors associated with pain in spinal cord injury: a systematic review and meta-analysis
Source: eClinicalMedicine. 2026 May 18;96:103976. doi: 10.1016/j.eclinm.2026.103976 (PMC13316355; doi:10.1016/j.eclinm.2026.103976)
Supplement: Appendix 6 - Publication Bias [file mmc6.docx]

**Appendix 6 – Publication Bias**

***Publication Bias Assessment***

**eTable 8.** **Overview of publication bias assessment for each meta-analysis**, including visual inspection funnel plots, Egger’s test *p*-values, the trim-and-fill method estimates, and adjusted pooled effect size (where applicable).

|  | **Funnel plot** | **Egger’s test** | **Trim-and-fill method** | **Adjusted pooled effect size** |
| --- | --- | --- | --- | --- |
| **Mental Health Factors** |  |  |  |  |
| **Depression (k = 49)** | Symmetrical | z = 0.42, *p* = 0.67 | *No missing studies imputed* | *No change* |
| **Anxiety (k = 23)** | Symmetrical | z = 1.23, *p* = 0.22 | *No missing studies imputed* | *No change* |
| **Psychological Health (k = 18)** | Symmetrical | z = -0.88, *p* = 0.38 | *No missing studies imputed* | *No change* |
| **Adaptive Psychological Factors** |  |  |  |  |
| **Self-efficacy (k = 16)** | Symmetrical | z = 0.07, *p* = 0.94 | **One study imputed** | *No change* |
| **Acceptance (k = 9)** | Symmetrical | z = -0.11, *p* = 0.91 | *No missing studies imputed* | *No change* |
| **Resilience (k = 5)** | Symmetrical | z = -1.24, *p* = 0.21 | **One study imputed** | *r* = -0.26, 95% CI [-0.41, -0.09] |
| **Cognitive and Emotional Factors** |  |  |  |  |
| **Catastrophising (k = 18)** | Symmetrical | z = -0.20, *p* = 0.84 | **Three studies imputed** | *r* = 0.41, 95% CI [0.33, 0.49] |
| **Stress (k = 6)** | Symmetrical | z = 0.20, *p* = 0.84 | **Two studies imputed** | *r* = 0.17, 95% CI [0.08, 0.25] |
| **Anger (k = 5)** | **Asymmetrical** | **z = 3.56, *p* < 0.001** | *No missing studies imputed* | *No change* |
| **Social and Interpersonal Factors** |  |  |  |  |
| **Perceived Social Functioning (k = 9)** | Symmetrical | z = -0.30, *p* = 0.76 | **One study imputed** | *r* = -0.27, 95% CI [-0.33, -0.21] |
| **Social Integration (k = 7)** | Symmetrical | z = 0.04, *p* = 0.97 | *No missing studies imputed* | *No change* |
| **Social Support (k = 6)** | Symmetrical | z = 1.33, *p* = 0.18 | **Two studies imputed** | *r* = -0.18, 95% CI [-0.42, 0.09] |
| **Fatigue and Sleep** |  |  |  |  |
| **Fatigue (k = 10)** | Symmetrical | z = -0.07, *p* = 0.95 | *No missing studies imputed* | *No change* |
| **Sleep disturbance (k = 9)** | Symmetrical | z = 0.51, *p* = 0.61 | **Two studies imputed** | ***r* = 0.16, 95% CI [-0.06, 0.36]** |
| **Quality of Life and Life Satisfaction** |  |  |  |  |
| **Quality of life and life satisfaction (k = 16)** | Symmetrical | z = -0.98, *p* = 0.33 | **Three studies imputed** | *r* = -0.26, 95% CI [-0.34, -0.18] |

***Funnel Plots***


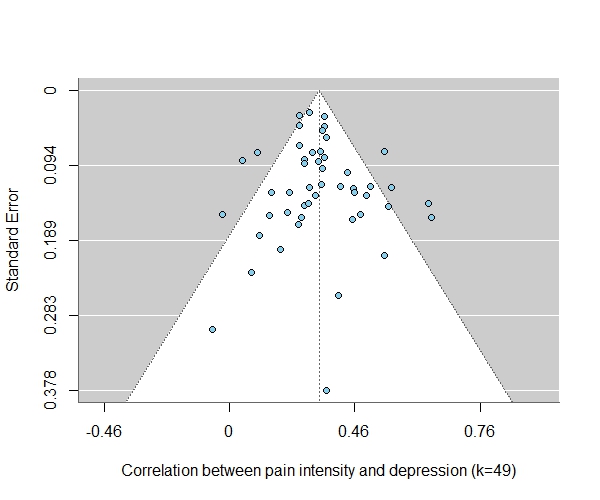


**eFigure 9.** **Funnel plot for the meta-analysis of pain–depression association across 49 studies.** Each point represents an individual study’s effect size (*r* value) plotted against its standard error. The funnel shape illustrates expected sampling variation around the pooled effect size (vertical line).


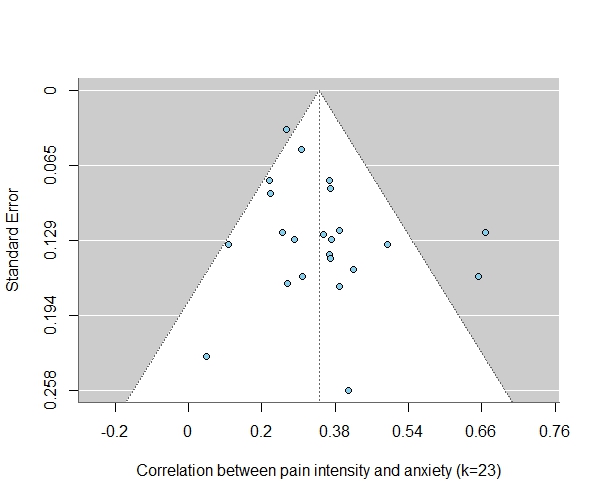


**eFigure 10.** **Funnel plot for the meta-analysis of pain–anxiety association across 23 studies.**


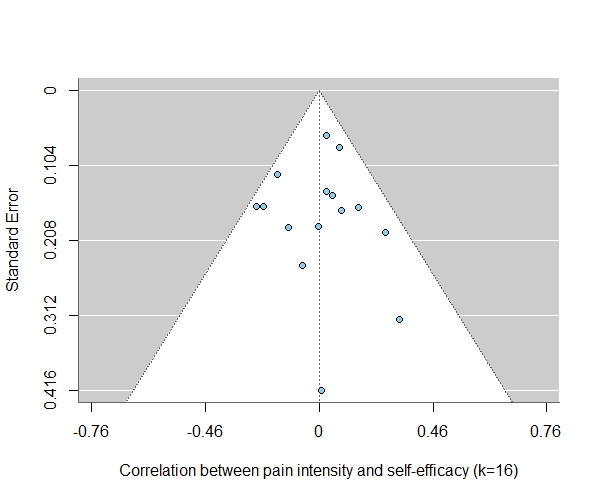


**eFigure 11.** **Funnel plot for the meta-analysis of association between pain and self-efficacy across 16 studies.**


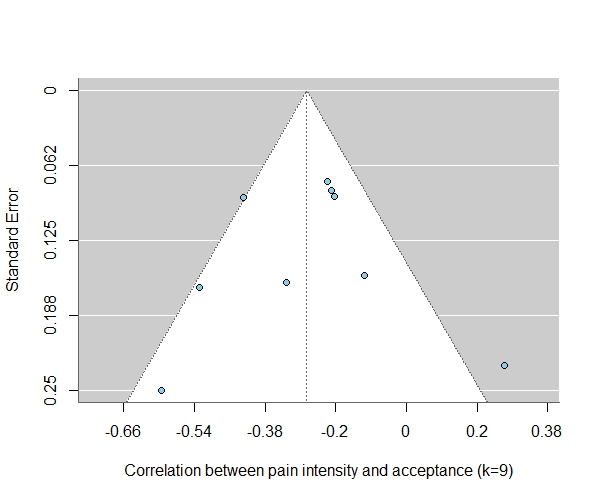


**eFigure 12.** **Funnel plot for the meta-analysis of pain–acceptance association across nine studies.**


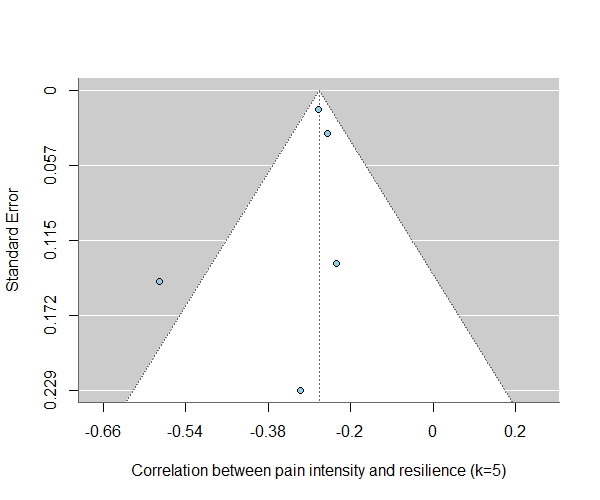


**eFigure 13.** **Funnel plot for the meta-analysis of pain–resilience association across five studies.**


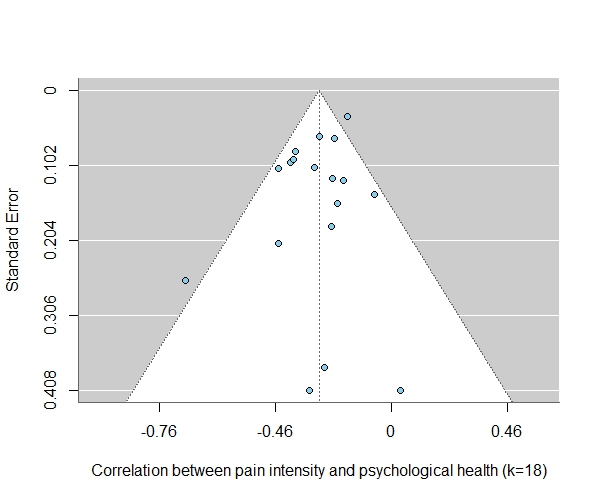


**eFigure 14.** **Funnel plot for the meta-analysis of pain–psychological health association across 18 studies.**


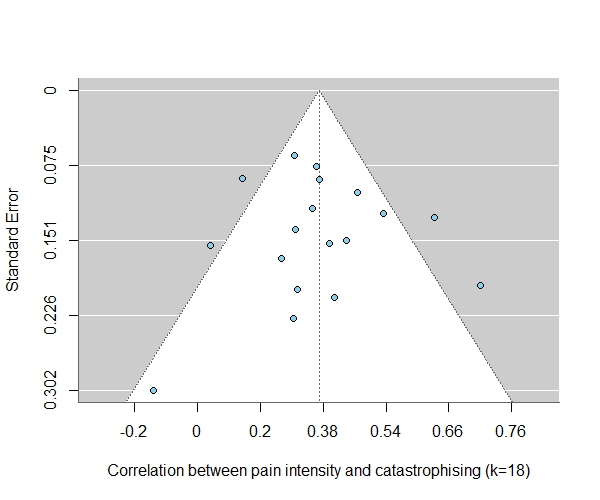


**eFigure 15.** **Funnel plot for the meta-analysis of pain–catastrophising association across 18 studies.**


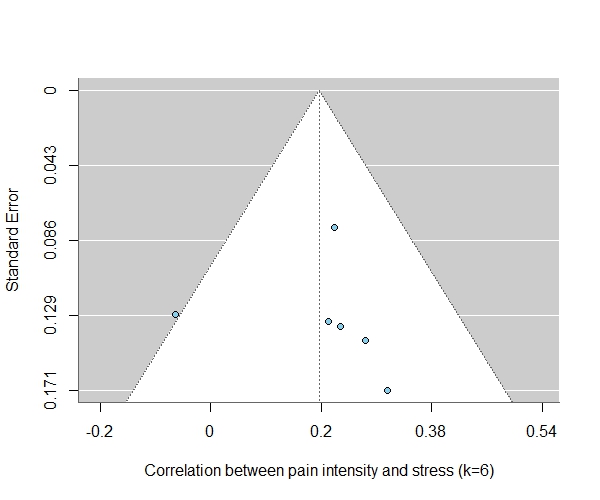


**eFigure 16.** **Funnel plot for the meta-analysis of pain–stress association across six studies.**


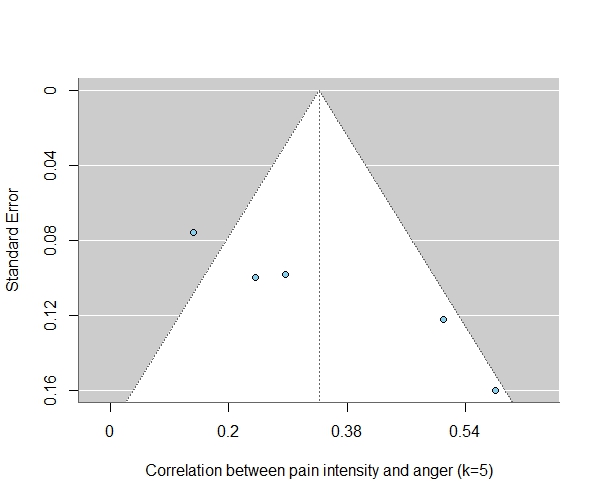


**eFigure 17.** **Funnel plot for the meta-analysis of pain–anger association across five studies.**


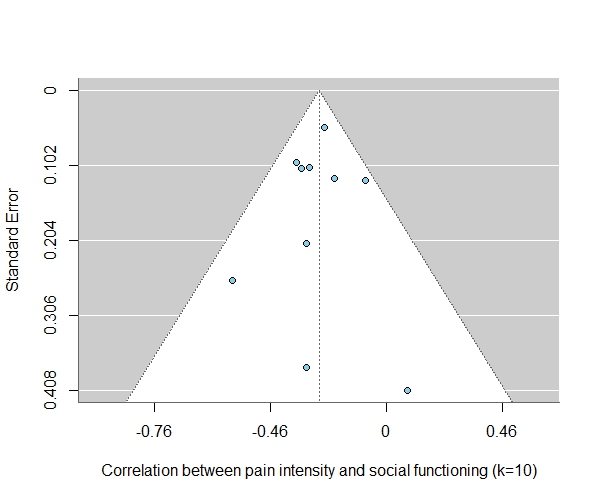


**Figure 18.** **Funnel plot for the meta-analysis of pain–social functioning association across nine studies.**


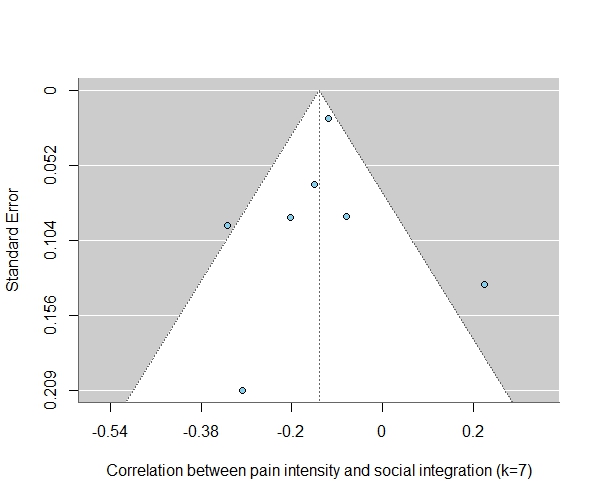


**eFigure 19.** **Funnel plot for the meta-analysis of pain–social integration association across seven studies.**


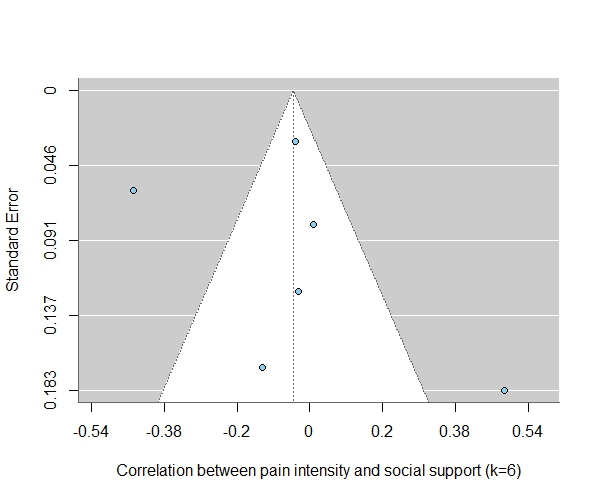


**eFigure 20.** **Funnel plot for the meta-analysis of pain–social support association across five studies.**


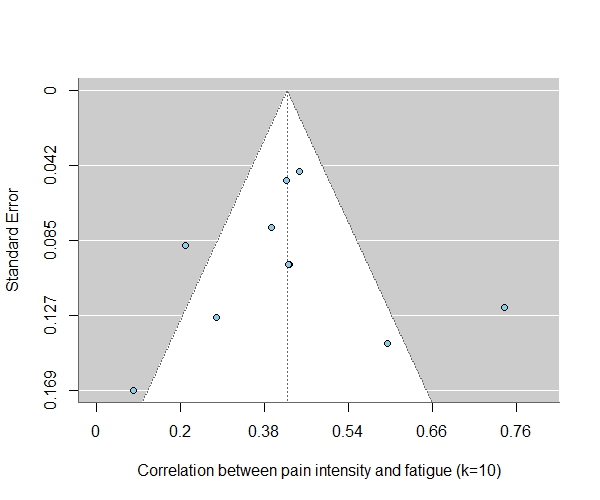


**eFigure 21.** **Funnel plot for the meta-analysis of pain–fatigue association across 10 studies.**


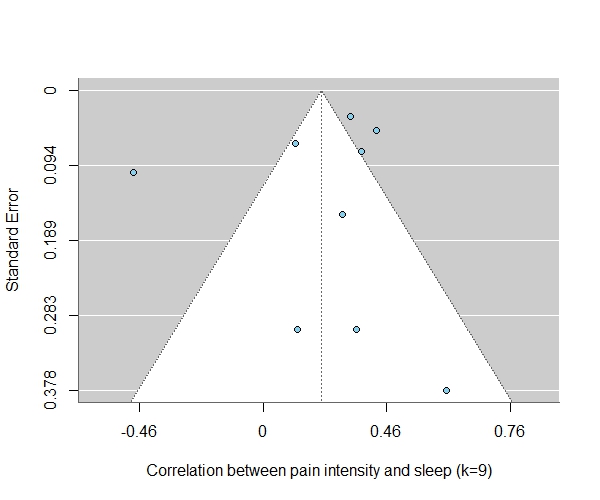


**eFigure 22.** **Funnel plot for the meta-analysis of pain–sleep association across nine studies.**


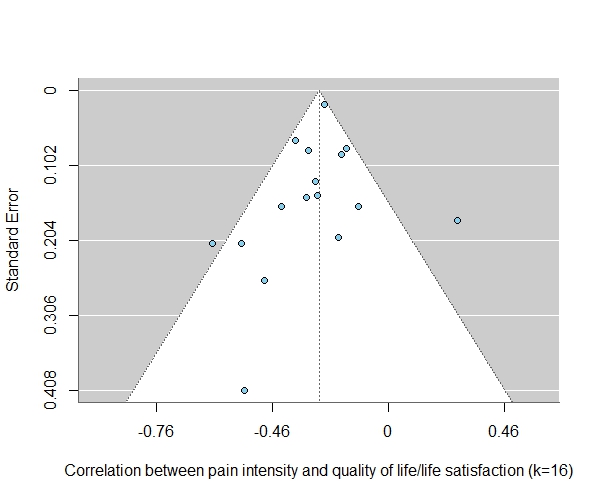


**eFigure 23.** **Funnel plot for the meta-analysis of association between pain and quality of life/life satisfaction across 16 studies.**
